# Coverage and Equity of Childhood Vaccines in China

**DOI:** 10.1001/jamanetworkopen.2022.46005

**Published:** 2022-12-09

**Authors:** Haijun Zhang, Xiaozhen Lai, Joshua Mak, Salin Sriudomporn, Haonan Zhang, Hai Fang, Bryan Patenaude

**Affiliations:** 1 Department of Health Policy and Management, School of Public Health, Peking University, Beijing, China; 2 China Center for Health Development Studies, Peking University, Beijing, China; 3 Department of International Health, Johns Hopkins Bloomberg School of Public Health, Baltimore, Maryland; 4 International Vaccine Access Center, Johns Hopkins Bloomberg School of Public Health, Baltimore, Maryland; 5 Peking University Health Science Center, Chinese Center for Disease Control and Prevention Joint Research Center for Vaccine Economics, Beijing, China; 6 Institute for Global Health and Development, Peking University, Beijing, China

## Abstract

**Importance:**

Measuring vaccination coverage rates and equity is crucial for informing immunization policies in China.

**Objectives:**

To estimate coverage rates and multidimensional equity for childhood vaccination in China.

**Design, Setting, and Participants:**

This cross-sectional study was conducted via a survey in 10 Chinese provinces between August 5 and October 16, 2019, among children ages 6 months to 5 years and their primary caregivers. Children’s vaccination records and their primary caregivers’ demographics and socioeconomic status were collected. Data were analyzed from November 2019 to March 2022.

**Main Outcomes and Measures:**

Vaccine coverage rates were measured as a percentage of National Immunization Program (NIP) and non-NIP vaccines administered before the day on which the child was surveyed. A multidimensional equity model applied a standardized approach to ranking individuals from least to most unfairly disadvantaged by estimating differences between observed vaccination status and estimated vaccination status as function of fair and unfair variation. Fair sources of variation in coverage included whether the child was of age to receive the vaccine, and unfair sources of variation included sex of the child and sociodemographic characteristics of caregivers. Absolute equity gaps (AEGs), concentration index values, and decompositions of factors associated with vaccine equity were estimated in the model.

**Results:**

Vaccine records and sociodemographic information of 5294 children (2976 [52.8%] boys and 2498 [47.2%] girls; age range, 6-59 months; 1547 children aged 12-23 months) and their primary caregivers were collected from 10 provinces. Fully immunized coverage under the NIP was 83.1% (95% CI, 82.0%-84.1%) at the national level and more than 80% in 7 provinces (province coverage ranged from 77.8% [95% CI, 74.3% to 81.3%] in Jiangxi to 88.4% [95% CI, 85.7%-91.1%] in Beijing). For most non-NIP vaccines, however, coverage rates were less than 50%, ranging from 1.8% (95% CI, 1.3%-2.2%) for the third dose of rotavirus vaccine to 67.1% (65.4% to 68.8%) for the first dose of the varicella vaccine. The first dose of *Haemophilus influenzae* type b vaccine had the largest AEG, at 0.603 (95% CI, 0.570-0.636), and rotavirus vaccine dose 3 had the largest concentration index value, at 0.769 (95% CI, 0.709-0.829). The largest share of non-NIP vaccine inequity was contributed by monthly family income per capita, followed by education level, place of residence, and province for caregivers. For example, the proportion of explained inequity for pneumococcal conjugate vaccine dose 3 was 40.94% (95% CI, 39.49%-42.39%), 22.67% (95% CI, 21.43%-23.9%), 27.15% (95% CI, 25.84%-28.46%), and 0.68% (95% CI, 0.44%-0.92%) for these factors, respectively.

**Conclusions and Relevance:**

This cross-sectional study found that NIP vaccination coverage in China was high but there was inequity for non-NIP vaccines. These findings suggest that improvements in equitable coverage of non-NIP vaccination may be urgently needed to meet national immunization goals.

## Key Points

**Question:**

What are the rates and equity levels of childhood vaccination in China?

**Findings:**

In this cross-sectional study of vaccination records and socioeconomic status among 5294 children and their primary caregivers in China, coverage rates for National Immunization Program (NIP) vaccines were higher than those for non-NIP vaccines. There was significant inequity among non-NIP vaccines.

**Meanings:**

This study’s findings may provide reliable estimates of childhood vaccine coverage rates and equity for NIP and non-NIP vaccines in China from a multidimensional equity model.

## Teaser

This cross-sectional study investigates coverage rates and equity of childhood vaccines in China in 2019.

## Introduction

Vaccination is one of the most successful and effective public health interventions in history, saving countless lives from various infectious diseases.^1,2,3^ The Expanded Program on Immunization (EPI) contributed significantly to reducing the global burden of vaccine-preventable diseases among children.^4,5,6^ The successor of EPI, the 2011 to 2020 Global Vaccine Action Plan (GVAP), was launched in 2012 by the World Health Assembly.^7^ It included various targets for childhood vaccination, such as 90% country-level coverage of all vaccines in EPI and equitable access to vaccination for everyone by 2020.

In China, EPI has been a cornerstone health policy.^8^ According to the 2019 Vaccine Administration Law of the People’s Republic of China,^9^ vaccines were divided into 2 categories: National Immunization Program (NIP) and non-NIP vaccines. The government provided NIP vaccines free of charge to citizens, and non-NIP vaccines were voluntarily administered and self-funded by adults or caregivers of children. Now, China aims to have coverage rates for NIP vaccines of more than 90% at the township level.^10^

Registration-based administrative reports for NIP vaccines have indicated coverage rates of more than 90% and even 99% at the national level.^11^ Administrative estimates, however, may be inaccurate owing to an incomplete birth registry data used as denominators, unreliable vaccination records, and delayed or duplicate reporting.^12,13^ Population-representative surveys collecting vaccine coverage information in multiple settings worldwide included the Demographic and Health Survey^14^ and large-scale Multiple Indicators Cluster Survey^15^; however, these studies were not available for China. Reliable coverage rates of NIP and non-NIP vaccines are key indicators needed in estimation models used for calculating the burden of infectious disease in China.^16^ This study aimed to estimate coverage rates and multidimensional equity for childhood vaccination in China.

## Methods

This cross-sectional study was ethically reviewed and approved by the Peking University Institutional Review Board. Written informed consent was obtained from survey respondents. This study is reported following the Strengthening the Reporting of Observational Studies in Epidemiology (STROBE) reporting guideline.

### Study Design

We conducted a cross-sectional survey of 10 provinces and municipalities to collect vaccination records of children in China in 2019. We used a multistage sampling scheme. First, according to the 5 layers of health expenditure responsibility between central and local governments stratified by the State Council in 2018 (eFigure 1 in Supplement 1),^17^ we chose 10 provinces and municipalities (3 in the first layer, 3 in the second layer, 1 in the third layer, 1 in the fourth layer, and 1 in the fifth layer) for our survey in terms of their socioeconomic development levels and locations. Second, we chose a developed city or district and a less developed city or district in each province or municipality. Third, we chose 2 subdistricts or counties in each city or district in which 3 or more community health centers or township clinics as vaccination sites were randomly selected to participate in our survey. The final study setting included 148 vaccination sites in 10 provinces or municipalities (34 counties or districts in 17 cities). The sample size calculation used for this survey is described in eMethods 1 in Supplement 1.

For 148 randomly selected vaccination centers on surveyed dates, caregivers who brought their children for NIP vaccination and agreed to participate in this study were interviewed using portable tablet devices (Samsung). We designed questionnaires with automatic logical proofreading for reducing missing values and errors. Questionnaires collected participants’ and their children’s sociodemographic information, including age, birth date, sex, education level, monthly family income per capita, place of residence (urban or rural), province, and health insurance coverage type. Specific categorizations and units of variables are reported in Table 1. Among 6668 respondents, 5294 individuals (79.4%) provided complete vaccination records. The interviewer took photos to document the date and types of each vaccine that were received. A flow chart of each stage of participant recruitment is shown in eFigure 2 in Supplement 1. We report information on missing data in eTable 1 in Supplement 1.

**Table 1.** Characteristics of Respondents and Children

Characteristic	Participants, No. (%)										
	Province										National (n = 5294)
	Beijing (n = 551)	Chongqing (n = 567)	Gansu (n = 390)	Guangdong (n = 511)	Henan (n = 516)	Jiangxi (n = 554)	Jilin (n = 613)	Shandong (n = 522)	Shanghai (n = 525)	Yunnan (n = 545)	
**Children**											
Age, mo											
6-11	134 (24.3)	175 (30.9)	116 (29.7)	132 (25.8)	147 (28.5)	162 (29.2)	174 (28.4)	140 (26.8)	146 (27.8)	113 (20.7)	1439 (27.2)
12-23	197 (35.8)	156 (27.5)	95 (24.4)	148 (29.0)	140 (27.1)	166 (30.0)	210 (34.3)	157 (30.1)	126 (24.0)	152 (27.9)	1547 (29.2)
24-35	112 (20.3)	95 (16.8)	86 (22.1)	85 (16.6)	80 (15.5)	96 (17.3)	87 (14.2)	121 (23.2)	96 (18.3)	123 (22.6)	981 (18.5)
36-59	108 (19.6)	141 (24.9)	93 (23.9)	146 (28.6)	149 (28.9)	130 (23.5)	142 (23.2)	104 (19.9)	157 (29.9)	157 (28.8)	1327 (25.1)
Sex											
Boys	294 (53.4)	294 (51.9)	200 (51.3)	280 (54.8)	275 (53.3)	281 (50.7)	319 (52.0)	264 (50.6)	284 (54.1)	305 (55.0)	2796 (52.8)
Girls	257 (46.6)	273 (48.1)	190 (48.7)	231 (45.2)	241 (46.7)	273 (49.3)	294 (48.0)	258 (49.4)	241 (45.9)	240 (44.0)	2498 (47.2)
**Respondents**											
Age, y											
<30	117 (21.2)	203 (35.8)	115 (29.5)	177 (34.6)	198 (38.4)	152 (27.4)	200 (32.6)	117 (22.4)	141 (26.9)	217 (39.8)	1637 (30.9)
30-39	318 (57.7)	170 (30.0)	165 (42.3)	233 (45.6)	236 (45.7)	243 (43.9)	303 (49.4)	293 (56.1)	279 (53.1)	217 (39.8)	2457 (46.4)
40-49	38 (6.9)	70 (12.4)	28 (7.2)	46 (9.0)	21 (4.1)	47 (8.5)	47 (7.7)	65 (12.5)	41 (7.8)	55 (10.1)	458 (8.7)
≥50	78 (14.2)	124 (21.9)	82 (21.0)	55 (10.8)	61 (11.8)	112 (20.2)	63 (10.3)	47 (9.0)	64 (12.2)	56 (10.3)	742 (14.0)
Family relationship to child											
Mother	349 (63.3)	337 (59.4)	235 (60.3)	362 (70.8)	366 (70.9)	350 (63.2)	399 (65.1)	402 (77.0)	354 (67.4)	380 (69.7)	3534 (66.8)
Father	117 (21.2)	74 (13.1)	61 (15.6)	91 (17.8)	83 (16.1)	75 (13.5)	138 (22.5)	72 (13.8)	98 (18.7)	98 (18.0)	907 (17.1)
Grandparent	85 (15.4)	156 (27.5)	94 (24.1)	58 (11.4)	67 (13.0)	129 (23.3)	76 (12.4)	48 (9.2)	73 (13.9)	67 (12.3)	853 (16.1)
Education level											
≤Elementary school	15 (2.7)	122 (21.5)	58 (14.9)	43 (8.4)	39 (7.6)	81 (14.6)	46 (7.5)	11 (2.1)	12 (2.3)	94 (17.3)	521 (9.8)
Junior high school	73 (13.3)	143 (25.2)	106 (27.2)	176 (34.4)	135 (26.2)	155 (28)	195 (31.8)	109 (20.9)	96 (18.3)	167 (30.6)	1355 (25.6)
High school or vocational school	132 (23.9)	132 (23.3)	85 (21.8)	121 (23.7)	125 (24.2)	109 (19.7)	126 (20.6)	151 (28.9)	94 (17.9)	119 (21.8)	1194 (22.6)
Associate degree (2 y)	116 (21.1)	85 (15.0)	71 (18.2)	85 (16.6)	92 (17.8)	90 (16.3)	115 (18.8)	139 (26.6)	122 (23.2)	82 (15.1)	997 (18.8)
≥Bachelor’s degree	215 (39.0)	85 (15.0)	70 (18.0)	86 (16.8)	125 (24.2)	119 (21.5)	131 (21.4)	112 (21.5)	201 (38.3)	83 (15.2)	1227 (23.2)
Place of residence											
Rural	233 (42.3)	180 (31.8)	245 (62.8)	160 (31.3)	279 (54.1)	279 (50.4)	175 (28.6)	141 (27.0)	138 (26.3)	309 (56.7)	2139 (40.4)
Urban	318 (57.7)	387 (68.3)	145 (37.2)	351 (68.7)	237 (45.9)	275 (49.6)	438 (71.5)	381 (73.0)	387 (73.7)	236 (43.3)	3155 (59.6)
Quantile of monthly family income/mo/capita^a^											
1 (<¥1000)	54 (9.8)	183 (32.3)	143 (36.7)	112 (21.9)	124 (24.0)	85 (15.3)	160 (26.1)	67 (12.8)	13 (2.5)	211 (38.7)	1152 (21.8)
2 (¥1001-¥1600)	49 (8.9)	88 (15.5)	98 (25.1)	94 (18.4)	110 (21.3)	105 (19.0)	110 (17.9)	109 (20.9)	34 (6.5)	103 (18.9)	900 (17.0)
3 (¥1601-¥2400)	88 (16.0)	91 (16.1)	78 (20.0)	115 (22.5)	111 (21.5)	130 (23.5)	157 (25.6)	151 (28.9)	53 (10.1)	89 (16.3)	1063 (20.1)
4 (¥2401-¥3750)	147 (26.7)	118 (20.8)	54 (13.9)	96 (18.8)	108 (20.9)	133 (24.0)	116 (18.9)	143 (27.4)	137 (26.1)	80 (14.7)	1132 (21.4)
5 (>¥3751)	213 (38.7)	87 (15.3)	17 (4.4)	94 (18.4)	63 (12.2)	101 (18.2)	70 (11.4)	52 (10.0)	288 (54.9)	62 (11.4)	1047 (19.8)
Medical insurance type^a^											
Urban rural resident medical insurance	205 (37.2)	387 (68.3)	304 (78.0)	309 (60.5)	354 (68.6)	381 (68.8)	414 (67.5)	290 (55.6)	195 (37.1)	395 (72.5)	3234 (61.1)
Urban employee-based medical insurance	315 (57.2)	161 (28.4)	84 (21.5)	178 (34.8)	145 (28.1)	165 (29.8)	163 (26.6)	219 (42.0)	315 (60.0)	140 (25.7)	1885 (35.6)
None	31 (5.6)	19 (3.4)	2 (0.5)	24 (4.7)	17 (3.3)	8 (1.4)	36 (5.9)	13 (2.5)	15 (2.9)	10 (1.8)	175 (3.3)

^a^ ¥1 = $0.145 in 2019.

### Statistical Analysis

We applied a standardized model developed under the Vaccine Economics Research for Sustainability and Equity (VERSE) program at the Johns Hopkins University Bloomberg School of Public Health International Vaccine Access Center to calculate vaccination coverage and measure multidimensional vaccine equity.^18^ The composite assessment metric on vaccination equity in the VERSE model was derived from literature on the measurement of socioeconomic equity combined with social choice theory and measures of direct unfairness in health care access.^19,20,21,22,23^

To parameterize the VERSE model, fair and unfair sources of variation in vaccination status were identified and measured at the individual level. Fair sources of variation in coverage included whether the child surveyed was underage to receive a vaccine according to vaccination schedules in China (eTables 2 and 3 in Supplement 1). We used birth and survey dates to calculate the age of a child and compared it with the appropriate vaccination schedule to determine whether the child was underage. Unfair sources of variation in the model included sex of the child, caregiver education level, monthly family income per capita, health insurance coverage, place of residence (ie, urban vs rural), and province. These factors were chosen based on stakeholder engagement and near-universal data collection along these dimensions by national health information systems.^14^ Similar to a propensity score, the direct unfairness ranking metric was computed as the estimated probability of vaccination, holding fair determinants at reference levels and allowing unfair determinants to vary. This propensity for unfair disadvantage metric was then used as the ranking variable in a Wagstaff concentration index, along with the cumulative share of the outcome, to compute the composite coverage equity metric.^24^

Because the metric was based on Wagstaff concentration index, regression-based Kitagawa-Blinder-Oaxaca decomposition was used to generate the cumulative share of overall observed inequality contributed by fair and unfair factors.^20,25,26^ Finally, the AEG was derived from the concentration index. This involved subtracting the outcome from the top 20% of the study sample ranked by multidimensional unfairness and the bottom 20% of the study sample.

The standardized Wagstaff’s concentration index (*CI_direct_*) formula is:

CI_direct_ = 2Cov(hci_direct_, F(hci_du_))/μhc

The AEG formula is:

AEG = *hci_direct_* (top 20% (*F*(*hci_du_*))) − *hci_direct_* (bottom 20% (*F*(*hci_du_*)))

In this equation,* hci_direct_* = directly standardized individual level of health care (observed vaccination coverage),* F*(*hci_du_*) = the cumulative distribution function of direct unfairness, and *μhc* = mean level of health care (vaccination coverage) across the entire population.

The AEG was interpreted as the percentage point deviation of the health care outcome measured between the top 20% and bottom 20% of the population ranked by unfair disadvantage. Because this was equivalent to isolating the top and bottom quintiles of the standard (Wagstaff) concentration index, this value was accompanied by the decomposition corresponding to the Wagstaff concentration index to show the share of overall inequity contributed by each contributing factor.

A fundamental assumption of the VERSE model was that every child should be vaccinated by the recommended age in national immunization schedules. Therefore, the only source of fair variation in vaccination status would be the age of the child. This meant that children who were younger than the recommended age for a specific vaccination could fairly be expected not to have received a vaccination and should be subtracted from the unfair disadvantage metric computation process. Other factors associated with variation in vaccination status should be considered as unfair factors associated with vaccination coverage. Reference levels for factors in the analysis were set at the national level, so negative indices at the subnational level would signal a protective association between unfair risk factors and outcomes. Such negative values would indicate a prodisadvantaged distribution of vaccination within that geographic subunit with respect to national-level factors associated with disadvantage. Additional details of the VERSE calculation and modeling process are described in eMethods 2 in Supplement 1 and were published in a previous study.^18^ Data were analyzed from November 2019 to March 2022. Analyses were done using R statistical software version 4.1.0 (R Project for Statistical Computing).

Outcomes included vaccine coverage rates, absolute equity gap (AEG), concentration index, and the contribution of factors associated with vaccine equity. Zero dose was defined as never having received a dose of diphtheria-tetanus-pertussis vaccine, poliovirus vaccine (PV), or measles, mumps, and rubella vaccine by age 12 months. Being fully NIP vaccine immunized for age was defined as having received all recommended national immunization schedule vaccines appropriate for the age of the child observed.

## Results

We analyzed questionnaires and vaccination records from 5294 children (2976 [52.8%] boys and 2498 [47.2%] girls; age range, 6-59 months). Table 1 shows sociodemographic characteristics of respondents and their children. At the national level, there were 1439 children aged 6 to 11 months (27.2%), 1547 children aged 12 to 23 months (29.2%), 981 children aged 24 to 35 months (18.5%), and 1327 children aged 36 to 59 months (25.1%). Most respondents were parents (4441 respondents [83.9%]), and 4094 respondents (77.3%) were younger than age 40 years. There were 3418 respondents (64.6%) with a high school or higher education, 3155 respondents (59.6%) from an urban setting, and 2052 respondents (38.8%) with a monthly family income per capita less than ¥1600 (US $232). There were 175 respondents (3.3%) who did not have medical insurance. The distributions of respondent sex and place of residence in our survey sample were similar to those in the China Population and Employment Statistics Yearbook 2020.^27^

All NIP vaccine coverage rates were found to be more than 85% at the national level, with most at more than 90% (Table 2). The highest 3 NIP vaccine coverage rates nationally were the first dose of PV (99.8%; 95% CI, 99.7%-99.9%), the first dose of hepatitis B vaccine (hepB1; 99.4%; 95% CI, 99.2%-99.6%), and bacillus Calmette-Guérin vaccine (99.3%; 95% CI, 99.1%-99.5%). The lowest NIP vaccine coverage was observed for the second dose of Japanese encephalitis vaccine (JEV2; 87.7%; 95% CI, 85.5%-89.8%). The proportion of children who were fully NIP vaccine immunized for age was 83.1% (95% CI, 82.0%-84.1%). Within provinces, most NIP vaccine coverage rates were also higher than 90.0%; these ranged from 72.4% (95% CI, 63.6%-81.3%) for JEV2 in Jiangxi to 100% (95% CI, 100%-100%) for diphtheria and tetanus toxoids and acellular pertussis vaccine dose 1 (DTaP1) in Chongqi; PV1 in Jilin, Shangdong, and Shanghai; hepB1 in Beijing and Chongqi, and hepB3 in Gansu. Fully immunized coverage under the NIP was more than 80% in 7 provinces. The highest and lowest rates of fully NIP vaccine immunized for age were 88.4% (95% CI, 85.7%-91.1%) in Beijing and 77.8% (95%CI, 74.3%-81.3%) in Jiangxi. The proportion of children who were zero dose was 0 in 9 provinces (Table 2).

**Table 2.** Estimated Vaccination Coverage by Vaccine and Dose

Vaccine	Coverage by province, % (95% CI)										
	Beijing	Chongqing	Gansu	Guangdong	Henan	Jiangxi	Jilin	Shandong	Shanghai	Yunnan	National
**NIP vaccines**											
BCG	99.6 (99.1 to 100)	99.5 (98.9 to 100)	99.7 (99.2 to 100)	99.0 (98.2 to 99.9)	99.2 (98.5 to 100)	99.5 (98.8 to 100)	99.2 (98.5 to 99.9)	99.8 (99.4 to 100)	98.5 (97.4 to 99.5)	99.1 (98.3 to 99.9)	99.3 (99.1 to 99.5)
DTaP1	99.2 (98.5 to 100)	100 (100 to 100)	98.3 (97 to 99.7)	97.5 (96.2 to 98.9)	99.0 (98.1 to 99.9)	99.6 (99.1 to 100)	99.8 (99.5 to 100)	99.6 (99.0 to 100)	99.4 (98.7 to 100)	98.7 (97.7 to 99.6)	99.2 (98.9 to 99.4)
DTaP3	97.5 (96.0 to 98.9)	96.2 (94.4 to 98)	95.6 (93.2 to 97.9)	92.8 (90.3 to 95.3)	94.6 (92.5 to 96.8)	93.5 (91.2 to 95.8)	99.6 (99.0 to 100)	95.3 (93.2 to 97.3)	95.5 (93.5 to 97.5)	95.1 (93.0 to 97.1)	95.6 (95.0 to 96.2)
PV1	99.6 (99.1 to 100)	99.8 (99.5 to 100)	99.7 (99.2 to 100)	99.6 (99.1 to 100)	99.4 (98.7 to 100)	99.8 (99.5 to 100)	100 (100 to 100)	100 (100 to 100)	100 (100 to 100)	99.8 (99.5 to 100)	99.8 (99.7 to 99.9)
PV2	99.0 (98.1 to 99.9)	98.4 (97.3 to 99.5)	99.1 (98.1 to 100)	99.2 (98.3 to 100)	98.5 (97.4 to 99.6)	99.2 (98.5 to 100)	100 (100 to 100)	98.5 (97.4 to 99.6)	99.2 (98.4 to 100)	99.4 (98.7 to 100)	99.1 (98.8 to 99.3)
PV3	97.6 (96.2 to 99)	96.4 (94.7 to 98.2)	98.7 (97.5 to 100)	98.4 (97.2 to 99.6)	96.8 (95.2 to 98.5)	97.1 (95.5 to 98.6)	99.4 (98.8 to 100)	95.4 (93.5 to 97.4)	98.2 (97.0 to 99.4)	97.4 (96.0 to 98.9)	97.6 (97.1 to 98)
HepB1	100 (100 to 100)	100 (100 to 100)	99.5 (98.8 to 100)	98.6 (97.6 to 99.6)	98.6 (97.6 to 99.6)	99.8 (99.5 to 100)	99.7 (99.2 to 100)	99.8 (99.4 to 100)	98.1 (96.9 to 99.3)	99.4 (98.8 to 100)	99.4 (99.2 to 99.6)
HepB3	99.3 (98.5 to 100)	98.5 (97.4 to 99.7)	100 (100 to 100)	96.7 (95.0 to 98.5)	98.5 (97.3 to 99.7)	98.8 (97.8 to 99.8)	98.5 (97.4 to 99.6)	97.8 (96.4 to 99.2)	99.0 (98.0 to 100)	98.0 (96.7 to 99.3)	98.5 (98.1 to 98.9)
JEV1	94.9 (92.8 to 97.0)	99.0 (97.9 to 100)	95.2 (92.7 to 97.8)	95.8 (93.8 to 97.8)	96.8 (95.0 to 98.6)	96.9 (95.2 to 98.6)	97.2 (95.7 to 98.8)	96.4 (94.5 to 98.2)	99.5 (98.7 to 100)	97.7 (96.3 to 99.1)	97.0 (96.4 to 97.5)
JEV2	86.9 (78.4 to 95.4)	90.5 (84.6 to 96.4)	96.6 (92.0 to 100)	84.3 (77.4 to 91.1)	86.8 (79.9 to 93.8)	72.4 (63.6 to 81.3)	93.0 (87.6 to 98.4)	92.1 (86 to 98.2)	88 (81.6 to 94.4)	90.4 (85.2 to 95.6)	87.7 (85.5 to 89.8)
MPSV-A1	98.2 (96.9 to 99.4)	97.4 (95.9 to 98.9)	97.7 (96.0 to 99.4)	94.5 (92.3 to 96.7)	95.1 (93.0 to 97.1)	97.3 (95.8 to 98.8)	97.3 (95.9 to 98.8)	97.2 (95.6 to 98.7)	98.5 (97.3 to 99.7)	96.4 (94.8 to 98.1)	96.9 (96.4 to 97.5)
MPSV-A2	97.4 (95.8 to 99.1)	91.9 (89 to 94.8)	96.5 (94.2 to 98.7)	88.3 (84.8 to 91.7)	89.9 (86.8 to 93.1)	89.3 (86.1 to 92.5)	92.3 (89.7 to 95.0)	80.3 (76.2 to 84.5)	96.4 (94.4 to 98.4)	92.2 (89.5 to 94.8)	91.3 (90.4 to 92.2)
MPSV-AC1	78.6 (57.1 to 100)	97.3 (92.1 to 100)	90.0 (76.9 to 100)	93.6 (86.6 to 100)	100 (100 to 100)	91.2 (81.6 to 100)	94.1 (82.9 to 100)	96.3 (91.3 to 100)	85.5 (76.1 to 94.8)	93.0 (86.4 to 99.6)	93.1 (90.6 to 95.6)
MMR1	99.3 (98.4 to 100)	99.5 (98.8 to 100)	98.2 (96.6 to 99.8)	98.2 (96.8 to 99.5)	98.4 (97.1 to 99.7)	99.2 (98.4 to 100)	96.5 (94.8 to 98.3)	99.7 (99.2 to 100)	99.7 (99.2 to 100)	98.6 (97.5 to 99.7)	98.7 (98.4 to 99.1)
HepA1	91.7 (88.1 to 95.3)	94.0 (91.1 to 96.9)	94.2 (90.9 to 97.5)	90.4 (86.8 to 94.1)	91.5 (88.0 to 95.1)	87.9 (83.8 to 92)	87.8 (83.6 to 91.9)	96.7 (94.4 to 98.9)	98.8 (97.5 to 100)	93.7 (90.9 to 96.4)	92.7 (91.6 to 93.7)
Zero^a^	0 (0 to 0)	0 (0 to 0)	0 (0 to 0)	0 (0 to 0)	0 (0 to 0)	0 (0 to 0)	0 (0 to 0)	0 (0 to 0)	0 (0 to 0)	0.2 (0 to 0.7)	0 (0 to 0.1)
NIP full^b^	88.4 (85.7 to 91.1)	86.4 (83.6 to 89.2)	85.4 (81.9 to 88.9)	78.3 (74.7 to 81.9)	80.8 (77.4 to 84.2)	77.8 (74.3 to 81.3)	86.3 (83.6 to 89)	78.5 (75.0 to 82.1)	85.7 (82.7 to 88.7)	82.6 (79.4 to 85.8)	83.1 (82.0 to 84.1)
**Non-NIP vaccines**											
PCV1	10.2 (7.6 to 12.8)	8.9 (6.5 to 11.4)	3.7 (1.7 to 5.7)	7.7 (5.3 to 10.1)	8.0 (5.6 to 10.5)	6.5 (4.4 to 8.5)	1.2 (0.3 to 2.1)	1.8 (0.6 to 3.0)	24.9 (21.1 to 28.7)	4.0 (2.3 to 5.7)	7.7 (6.9 to 8.4)
PCV3	6.9 (4.5 to 9.2)	6.9 (4.5 to 9.2)	2.2 (0.6 to 3.8)	4.4 (2.5 to 6.3)	4.6 (2.6 to 6.6)	3.9 (2.1 to 5.6)	0.8 (0 to 1.5)	1.1 (0.1 to 2.1)	18.9 (15.2 to 22.6)	2.3 (1.0 to 3.6)	5.1 (4.5 to 5.8)
Hib1	11.6 (8.9 to 14.4)	49.8 (45.5 to 54.1)	19.7 (15.6 to 23.9)	39.8 (35.4 to 44.1)	66.7 (62.6 to 70.9)	58.3 (54.1 to 62.5)	11.6 (9.0 to 14.2)	42 (37.6 to 46.3)	71.7 (67.7 to 75.7)	53.4 (49.2 to 57.7)	42.6 (41.3 to 44.0)
Hib3	5.3 (3.2 to 7.3)	31.9 (27.5 to 36.3)	8.9 (5.7 to 12.0)	16.5 (12.9 to 20.1)	46.4 (41.7 to 51.2)	35.2 (30.8 to 39.7)	1.4 (0.4 to 2.4)	20.1 (16.3 to 23.8)	56.8 (52.0 to 61.5)	29.2 (25.0 to 33.4)	25.0 (23.7 to 26.3)
Rota1	6.0 (4.0 to 8.0)	25.9 (22.3 to 29.6)	8.4 (5.6 to 11.2)	18.5 (15.2 to 21.9)	24.6 (20.8 to 28.3)	30.7 (26.8 to 34.5)	4.8 (3.1 to 6.5)	9.6 (7.1 to 12.1)	47.0 (42.7 to 51.3)	26.0 (22.3 to 29.7)	20.3 (19.2 to 21.3)
Rota3	0 (0 to 0)	0.9 (−0.1 to 1.8)	0 (0 to 0)	0.9 (−0.1 to 1.9)	0.3 (−0.3 to 0.9)	3.0 (1.2 to 4.8)	0 (0 to 0)	0 (0 to 0)	11.4 (7.7 to 15.0)	2.6 (1.0 to 4.2)	1.8 (1.3 to 2.2)
Vari1	64.4 (58.9 to 69.9)	72.4 (67.2 to 77.6)	26.1 (20.3 to 31.9)	60.1 (54.5 to 65.7)	80.8 (76.1 to 85.4)	63.6 (58.1 to 69.1)	53.1 (47.5 to 58.8)	76.1 (71.2 to 81.0)	95.3 (92.8 to 97.8)	71.8 (67.1 to 76.6)	67.1 (65.4 to 68.8)
EV71 (first)	21.2 (17.4 to 25.0)	49.5 (44.8 to 54.3)	15.2 (11.2 to 19.3)	42.4 (37.7 to 47.2)	50.7 (45.9 to 55.6)	54.6 (49.9 to 59.3)	24.8 (21.0 to 28.7)	32.6 (28.1 to 37.1)	60.5 (55.6 to 65.3)	39.7 (35.3 to 44.1)	39.5 (38.0 to 41.0)
EV71 (second)	20.5 (8.5 to 32.4)	18.3 (8.5 to 28.1)	16.7 (1.8 to 31.6)	24.6 (13.8 to 35.4)	17.0 (6.3 to 27.8)	22.4 (11.7 to 33.1)	9.3 (1.5 to 17.0)	18.2 (6.8 to 29.6)	42.0 (28.3 to 55.7)	44.6 (31.6 to 57.7)	23.9 (20.2 to 27.6)

Abbreviations: BCG, bacillus Calmette-Guérin vaccine; DTaP, diphtheria and tetanus toxoids and acellular pertussis vaccine; EV71, enterovirus 71 vaccine; hepA, hepatitis A vaccine; hepB, hepatitis B vaccine; Hib, *Haemophilus influenzae* type b vaccine; JEV, Japanese encephalitis vaccine; MMR, measles, mumps, and rubella vaccine; MPSV, meningococcal polysaccharide vaccine; NIP, National Immunization Program; NIP full, fully immunized for age; PCV, pneumococcal conjugate vaccine; PV, poliovirus vaccine; rota, rotavirus vaccine; vari, varicella vaccine; zero, zero dose.

^a^ Zero dose was defined as never having received a single dose of diphtheria-tetanus-pertussis vaccine, PV, or measles, mumps, and rubella vaccine by age 12 months.

^b^ Fully immunized for age was defined as having received all recommended NIP vaccines in the national immunization schedule appropriate for the age of the child observed.

Non-NIP vaccine coverage rates were less than 50%, except for the first dose of varicella vaccine (vari1) (Table 2). At the national level, the highest and lowest non-NIP vaccine coverage rates were 67.1% (95% CI, 65.4%-68.8%) for vari1 and 1.8% (95% CI, 1.3%-2.2%) for the third dose of rotavirus vaccine (rota3). Among all provinces and municipalities, the highest vaccine coverage for each non-NIP vaccine was in Shanghai, the most developed city in China.

We also estimated the AEG and concentration index values to capture equity in vaccine coverage rates at national and provincial levels (Table 3; eTable 4 in Supplement 1). We found that the AEG for NIP vaccines was small, with the AEG for each NIP vaccine less than 0.2 at the national level (ranging from 0.008; 95% CI 0.002-0.014 for PV1 to 0.193; 95% CI, 0.122-0.264 for JEV2). Conversely, AEGs for non-NIP vaccines were larger than those of NIP vaccines at the national level, suggesting that non-NIP vaccines were more inequitably distributed than NIP vaccines. The first dose of the *Haemophilus influenzae* type b (Hib1) vaccine had the largest AEG, at 0.603 (95% CI, 0.570-0.636), indicating that the most advantaged quintile had 60.3 percentage points higher coverage for this vaccine than the most disadvantaged quintile (eTable 4 in Supplement 1). The rota3 vaccine had the largest concentration index value, at 0.769 (95% CI, 0.709-0.829). Figure A shows coverage equity for NIP vaccines, with overall low levels of inequity within each province. For the third dose of pneumococcal conjugate vaccine (PCV3), Figure B shows that vaccine equity varied widely across provinces. Henan province had the highest degree of PCV3 equity, while Gansu and Yunnan had the largest levels of inequity. The equity coverage plane for all vaccines is presented in eFigure 3 in Supplement 1. Concentration index values showed similar results to AEGs for all vaccines, suggesting that much of the inequality was associated with differences between top and bottom quintiles. All NIP vaccines had small concentration indices compared with non-NIP vaccines. Among NIP vaccines, national concentration index values ranged from 0.044 (95% CI, 0.029-0.059) for JEV2 to 0.002 (95% CI, 0.001-0.003) for PV1. Values among non-NIP vaccines ranged from 0.769 (95% CI, 0.709-0.829) for rota3 to 0.132 (95% CI, 0.117 to 0.146) for vari1. Additionally, all non-NIP vaccinations had positive concentration indices. Among non-NIP vaccines, PCV, Hib and rota vaccines were the 3 most inequitably distributed according to AEG (PCV1: 0.231; 95% CI, 0.204-0.258; Hib1: 0.603; 95%CI, 0.570-0.636; rota1: 0.378; 95%CI, 0.347-0.409) and concentration index values (PCV3: 0.640; 95% CI, 0.598-0.682; Hib3: 0.430; 95% CI, 0.407- 0.453; rota3: 0.769; 95% CI, 0.709-0.829).

**Table 3.** Concentration Index Value by Vaccine and Dose

Vaccine	Concentration index value (95% CI)										
	Province										National
	Beijing	Chongqing	Gansu	Guangdong	Henan	Jiangxi	Jilin	Shandong	Shanghai	Yunnan	
**NIP vaccine**											
BCG	0.003 (−0.001 to 0.007)	0 (−0.004 to 0.003)	0.001 (−0.001 to 0.002)	0.007 (0 to 0.015)	0.001 (−0.002 to 0.004)	0.003 (−0.001 to 0.007)	0.001 (−0.002 to 0.004)	0.001 (−0.001 to 0.004)	0.004 (−0.001 to 0.01)	0.004 (−0.001 to 0.009)	0.003 (0.002 to 0.005)
DTaP1	−0.001 (−0.004 to 0.001)	0 (0 to 0)	0.006 (−0.005 to 0.017)	0.009 (0 to 0.019)	0.003 (−0.003 to 0.009)	0.003 (−0.001 to 0.008)	−0.001 (−0.004 to 0.001)	−0.001 (−0.003 to 0.001)	0 (−0.003 to 0.003)	0.004 (−0.003 to 0.010)	0.004 (0.003 to 0.006)
DTaP3	0.003 (−0.006 to 0.011)	0.002 (−0.010 to 0.015)	0.018 (0.004 to 0.032)	0.025 (0.009 to 0.041)	0.010 (−0.004 to 0.024)	0.026 (0.011 to 0.042)	−0.001 (−0.003 to 0.001)	0.016 (0.003 to 0.028)	0.001 (−0.009 to 0.012)	0.012 (0 to 0.025)	0.016 (0.012 to 0.020)
PV1	0.002 (−0.001 to 0.005)	0.001 (−0.001 to 0.003)	0.001 (−0.001 to 0.004)	0.003 (−0.001 to 0.007)	0.005 (−0.001 to 0.01)	0.001 (−0.001 to 0.004)	0 (0 to 0)	0 (0 to 0)	0 (0 to 0)	0.001 (−0.001 to 0.002)	0.002 (0.001 to 0.003)
PV2	0.005 (0 to 0.009)	0.009 (0.002 to 0.016)	0.008 (−0.001 to 0.016)	0.003 (−0.002 to 0.008)	0.009 (0.002 to 0.017)	0.005 (0 to 0.009)	0 (0 to 0)	0.008 (0.001 to 0.015)	0.004 (−0.002 to 0.010)	0.003 (−0.001 to 0.008)	0.006 (0.004 to 0.008)
PV3	0.009 (0.002 to 0.016)	0.012 (0 to 0.023)	0.007 (−0.002 to 0.016)	0.006 (−0.003 to 0.015)	0.010 (−0.001 to 0.022)	0.012 (0.003 to 0.021)	−0.002 (−0.006 to 0.002)	0.017 (0.004 to 0.031)	0.003 (−0.004 to 0.010)	0.006 (−0.004 to 0.016)	0.011 (0.008 to 0.014)
HepB1	0 (0 to 0)	0 (0 to 0)	0 (−0.005 to 0.005)	0.004 (−0.002 to 0.010)	0.001 (−0.005 to 0.008)	0.002 (−0.002 to 0.005)	0.002 (−0.001 to 0.005)	0.001 (−0.001 to 0.004)	0.006 (0 to 0.013)	0.003 (−0.001 to 0.006)	0.004 (0.002 to 0.005)
HepB3	0.004 (−0.001 to 0.008)	0.001 (−0.005 to 0.007)	0 (0 to 0)	0.010 (−0.001 to 0.021)	−0.001 (−0.006 to 0.005)	0.003 (−0.003 to 0.009)	0.008 (0.001 to 0.014)	0.006 (−0.003 to 0.015)	0.003 (−0.002 to 0.008)	0.004 (−0.003 to 0.011)	0.006 (0.004 to 0.008)
JEV1	0.017 (0.003 to 0.032)	−0.002 (−0.008 to 0.005)	0.021 (0.007 to 0.036)	0.005 (−0.009 to 0.019)	0.007 (−0.006 to 0.020)	0.002 (−0.008 to 0.012)	0.001 (−0.008 to 0.010)	0.004 (−0.01 to 0.017)	0.001 (−0.003 to 0.004)	0.004 (−0.005 to 0.014)	0.010 (0.006 to 0.013)
JEV2	0.020 (−0.042 to 0.082)	−0.003 (−0.045 to 0.038)	0.016 (−0.007 to 0.038)	0.029 (−0.012 to 0.07)	0.070 (0.021 to 0.119)	0.055 (−0.015 to 0.125)	0.011 (−0.017 to 0.040)	0.022 (−0.008 to 0.052)	0.003 (−0.032 to 0.037)	0.018 (−0.023 to 0.059)	0.044 (0.029 to 0.059)
MPSV-A1	0.002 (−0.005 to 0.010)	0.006 (−0.004 to 0.016)	0.009 (0 to 0.017)	0.017 (0.002 to 0.031)	0.010 (−0.005 to 0.025)	0.007 (−0.002 to 0.016)	0.003 (−0.007 to 0.012)	0.003 (−0.007 to 0.012)	0.001 (−0.008 to 0.010)	0.009 (0 to 0.019)	0.009 (0.006 to 0.013)
MPSV-A2	0.012 (0.001 to 0.024)	0.014 (−0.004 to 0.032)	0.006 (−0.011 to 0.022)	0.008 (−0.013 to 0.029)	0.013 (−0.008 to 0.034)	0.025 (0.005 to 0.044)	0.009 (−0.004 to 0.022)	0.032 (0.004 to 0.061)	−0.001 (−0.012 to 0.010)	0.014 (0 to 0.028)	0.031 (0.025 to 0.038)
MPSV-AC1	0.072 (−0.127 to 0.272)	0.022 (−0.021 to 0.066)	0.058 (−0.023 to 0.139)	0.036 (−0.006 to 0.079)	0 (0 to 0)	0.054 (−0.008 to 0.115)	0.012 (−0.021 to 0.044)	0.025 (−0.009 to 0.060)	0.044 (−0.022 to 0.111)	0.044 (0.001 to 0.086)	0.041 (0.025 to 0.057)
MMR1	0.002 (−0.002 to 0.006)	0.003 (−0.002 to 0.007)	0.010 (0 to 0.019)	0.010 (0.002 to 0.018)	0.008 (−0.001 to 0.016)	0.006 (−0.001 to 0.012)	0.007 (−0.001 to 0.016)	−0.001 (−0.003 to 0.001)	0 (0 to 0)	0.004 (−0.001 to 0.01)	0.007 (0.005 to 0.010)
HepA1	0.001 (−0.022 to 0.025)	0.005 (−0.016 to 0.026)	0.010 (−0.012 to 0.031)	0.020 (−0.002 to 0.042)	0.013 (−0.004 to 0.030)	−0.009 (−0.037 to 0.020)	0.047 (0.016 to 0.077)	0.009 (−0.004 to 0.023)	−0.008 (−0.018 to 0.002)	0.019 (0.003 to 0.035)	0.023 (0.017 to 0.030)
Zero^a^	NA	NA	NA	NA	NA	NA	NA	NA	NA	0.991 (0.980 to 1.002)	0.999 (0.998 to 1.000)
NIP full^b^	−0.002 (−0.019 to 0.015)	0.012 (−0.007 to 0.030)	0.037 (0.012 to 0.063)	0.011 (−0.015 to 0.037)	0.018 (−0.005 to 0.042)	0.036 (0.009 to 0.062)	0.005 (−0.013 to 0.022)	0.011 (−0.015 to 0.037)	0.009 (−0.011 to 0.028)	0.027 (0.004 to 0.049)	0.030 (0.023 to 0.038)
**Non-NIP vaccines**											
PCV1	0.507 (0.399 to 0.615)	0.496 (0.400 to 0.593)	0.737 (0.610 to 0.864)	0.635 (0.549 to 0.722)	0.246 (0.094 to 0.397)	0.418 (0.274 to 0.562)	0.294 (−0.083 to 0.672)	0.659 (0.496 to 0.823)	0.227 (0.147 to 0.307)	0.795 (0.734 to 0.855)	0.603 (0.568 to 0.637)
PCV3	0.607 (0.506 to 0.708)	0.485 (0.373 to 0.597)	0.878 (0.808 to 0.948)	0.669 (0.562 to 0.776)	0.157 (−0.058 to 0.371)	0.443 (0.270 to 0.617)	0.428 (0.027 to 0.829)	0.600 (0.445 to 0.756)	0.272 (0.171 to 0.372)	0.779 (0.693 to 0.865)	0.640 (0.598 to 0.682)
Hib1	0.393 (0.271 to 0.515)	0.148 (0.099 to 0.197)	0.606 (0.529 to 0.682)	0.131 (0.067 to 0.195)	0.043 (0.007 to 0.079)	−0.059 (−0.100 to −0.018)	0.091 (−0.029 to 0.212)	−0.030 (−0.090 to 0.031)	0.065 (0.032 to 0.097)	0.147 (0.101 to 0.192)	0.293 (0.276 to 0.309)
Hib3	0.567 (0.429 to 0.704)	0.275 (0.202 to 0.349)	0.678 (0.588 to 0.768)	0.454 (0.351 to 0.556)	0.153 (0.095 to 0.211)	−0.061 (−0.134 to 0.011)	0.237 (−0.110 to 0.584)	−0.150 (−0.262 to −0.039)	0.130 (0.082 to 0.179)	0.273 (0.194 to 0.351)	0.430 (0.407 to 0.453)
Rota1	0.471 (0.334 to 0.608)	0.336 (0.266 to 0.406)	0.584 (0.455 to 0.714)	0.099 (−0.012 to 0.209)	0.163 (0.076 to 0.249)	−0.110 (−0.183 to −0.038)	0.067 (−0.104 to 0.238)	−0.314 (−0.448 to −0.181)	0.089 (0.037 to 0.142)	0.214 (0.131 to 0.297)	0.381 (0.355 to 0.407)
Rota3	NA	0.503 (0.186 to 0.819)	NA	0.755 (0.562 to 0.948)	0.166 (0.048 to 0.284)	−0.024 (−0.403 to 0.354)	NA	NA	0.281 (0.117 to 0.446)	0.529 (0.301 to 0.758)	0.769 (0.709 to 0.829)
Vari1	0.033 (−0.017 to 0.082)	0.069 (0.028 to 0.110)	0.468 (0.374 to 0.562)	0.043 (−0.011 to 0.097)	0.025 (−0.008 to 0.059)	−0.061 (−0.110 to −0.011)	−0.033 (−0.095 to 0.028)	0.006 (−0.031 to 0.044)	−0.002 (−0.015 to 0.010)	0.041 (0.003 to 0.079)	0.132 (0.117 to 0.146)
EV71 (first)	−0.017 (−0.124 to 0.090)	0.163 (0.109 to 0.217)	0.573 (0.468 to 0.677)	0.129 (0.064 to 0.193)	0.053 (−0.003 to 0.108)	−0.042 (−0.092 to 0.008)	0.061 (−0.027 to 0.149)	−0.088 (−0.168 to −0.008)	0.057 (0.010 to 0.103)	0.204 (0.143 to 0.266)	0.221 (0.201 to 0.241)
EV71 (second)	−0.058 (−0.230 to 0.114)	0.248 (0.159 to 0.336)	0.715 (0.600 to 0.830)	0.216 (0.109 to 0.322)	0.138 (0.055 to 0.220)	−0.040 (−0.124 to 0.043)	0.094 (−0.126 to 0.315)	−0.038 (−0.178 to 0.103)	0.073 (−0.001 to 0.148)	0.270 (0.179 to 0.360)	0.311 (0.279 to 0.342)

Abbreviations: BCG, bacillus Calmette-Guérin vaccine; DTaP, diphtheria and tetanus toxoids and acellular pertussis vaccine; EV71, enterovirus 71 vaccine; hepA, hepatitis A vaccine; hepB, hepatitis B vaccine; Hib, *Haemophilus influenzae* type b vaccine; JEV, Japanese encephalitis vaccine; MMR, measles, mumps, and rubella vaccine; MPSV, meningococcal polysaccharide vaccine; NA, not applicable; NIP, National Immunization Program; NIP full, fully immunized for age; PCV, pneumococcal conjugate vaccine; PV, poliovirus vaccine; rota, rotavirus vaccine; vari, varicella vaccine; zero, zero dose.

^a^ Zero dose was defined as never having received a single dose of diphtheria-tetanus-pertussis vaccine, PV, or measles, mumps, and rubella vaccine by age 12 months.

^b^ Fully immunized for age was defined as having received all recommended NIP vaccines in the national immunization schedule appropriate for the age of the child observed.

**Figure.**
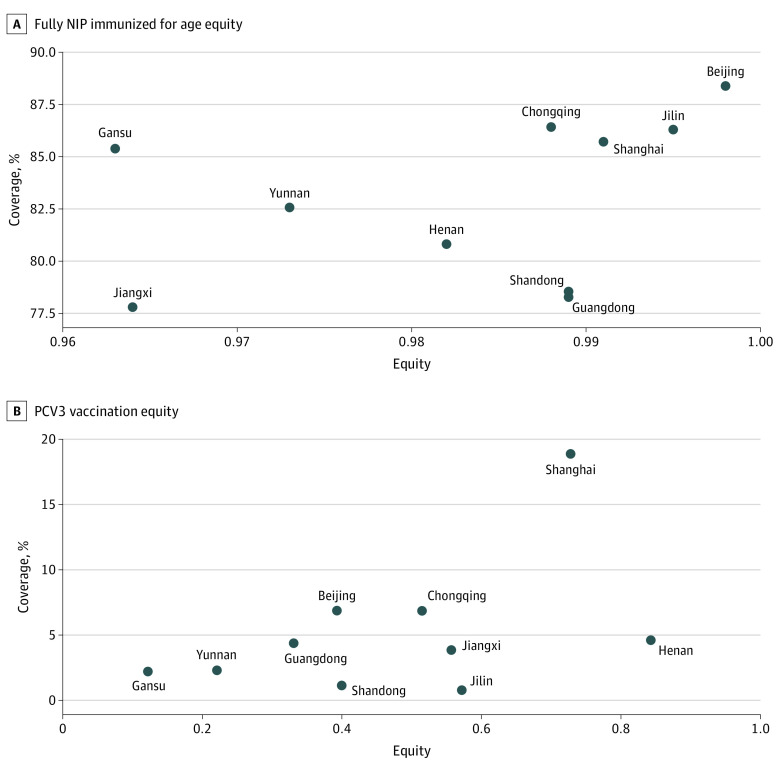
Equity Coverage Plane for 10 Provinces in China Fully National Immunization Program (NIP) vaccine immunized for age was defined as having received all recommended NIP vaccines in the national immunization schedule appropriate for the age of the child observed. Fully pneumococcal conjugate vaccine third dose (PCV3) immunized for age was defined as having received at least 3 doses of PCV. Equity = 1- composite index.

After decomposition of concentration index values over 7 measured factors hypothesized to be associated with equity, we found that 24.89% of observed inequality for being fully NIP vaccinated for age was explained by defined unfair sources of variation (unexplained variation for full NIP vaccination, 75.11%; 95% CI, 73.94%-76.27%) (Table 4). Factors with the greatest contribution to equity were sex of child and province, which could explain 8.49% (95% CI, 7.74%-9.29%) and 8.54% (95% CI, 7.74%-9.24%) of observed inequity, respectively. Decomposition figures for other vaccines are presented in eFigure 4 in Supplement 1, and decomposition proportions are shown in Table 4. Inequity found for most NIP vaccines was mainly contributed by unexplained variation and likely randomly distributed owing to the small contribution of unfair factors identified in the model. For example, 73.29% (95% CI, 71.94%-74.64%) of differences for DTaP3 could not be explained by unfair factors. For non-NIP vaccines, however, inequity could be largely explained by unfair factors, with monthly family income per capita, caregiver education level, place of residence, and province of caregiver contributing the most to observed inequality in coverage. For example, the proportion of explained inequity for PCV3 was 40.94% (95% CI, 39.49%-42.39%), 22.67% (95% CI, 21.43%-23.9%), 27.15% (95% CI, 25.84%-28.46%), and 0.68% (95% CI, 0.44%-0.92%) for these factors, respectively. The third dose of PCV had the largest proportion of equity (91.72%) explained by unfair factors among 5 non-NIP vaccines studied (unexplained variation, 8.28%; 95% CI, 7.46%-9.09%).

**Table 4.** Contribution of Factors Associated With Equity by Vaccine and Dose

Vaccine	Explained equity, % (95% CI)							
	Underage	Province	Urban vs rural residence	Caregiver education level	Monthly family income per capita	Sex	Medical insurance	Unexplained variation
**NIP vaccines**								
BCG	0 (0 to 0)	6.01 (5.37 to 6.66)	0.29 (0.15 to 0.44)	7.77 (7.05 to 8.5)	0.56 (0.36 to 0.76)	0.52 (0.32 to 0.71)	67.49 (66.22 to 68.75)	17.36 (16.34 to 18.38)
DTaP1	0 (0 to 0)	0.24 (0.10 to 0.37)	0.15 (0.04 to 0.26)	0.01 (−0.02 to 0.03)	0.75 (0.51 to 0.99)	4.25 (3.69 to 4.81)	1.44 (1.11 to 1.77)	93.17 (92.47 to 93.87)
DTaP3	0 (0 to 0)	0.40 (0.21 to 0.59)	12.78 (11.76 to 13.8)	3.61 (3.04 to 4.18)	9.18 (8.3 to 10.06)	0.74 (0.48 to 1.01)	0.01 (−0.02 to 0.03)	73.29 (71.94 to 74.64)
PV1	0 (0 to 0)	12.11 (11.23 to 13.00)	1.68 (1.33 to 2.03)	12.40 (11.51 to 13.29)	1.64 (1.29 to 1.98)	1.42 (1.1 to 1.74)	1.03 (0.75 to 1.3)	69.73 (68.48 to 70.97)
PV2	0 (0 to 0)	2.60 (2.15 to 3.05)	6.40 (5.7 to 7.09)	52.67 (51.26 to 54.08)	1.21 (0.9 to 1.52)	1.46 (1.12 to 1.8)	1.77 (1.39 to 2.14)	33.89 (32.56 to 35.23)
PV3	0 (0 to 0)	0.06 (−0.01 to 0.13)	12.77 (11.79 to 13.75)	23.17 (21.93 to 24.41)	6.12 (5.42 to 6.82)	0.80 (0.54 to 1.06)	0.55 (0.33 to 0.76)	56.54 (55.08 to 57.99)
HepB1	0 (0 to 0)	18.73 (17.68 to 19.78)	1.96 (1.59 to 2.33)	4.42 (3.86 to 4.97)	0.40 (0.23 to 0.56)	0.05 (−0.01 to 0.11)	0 (−0.01 to 0.02)	74.44 (73.27 to 75.62)
HepB3	0 (0 to 0)	3.41 (2.85 to 3.97)	6.23 (5.48 to 6.97)	1.54 (1.16 to 1.91)	0.69 (0.44 to 0.95)	2.79 (2.28 to 3.29)	62.99 (61.51 to 64.48)	22.35 (21.07 to 23.63)
JEV1	0 (0 to 0)	11.60 (10.59 to 12.62)	0.04 (−0.02 to 0.09)	0.75 (0.47 to 1.02)	0.04 (−0.02 to 0.10)	3.05 (2.51 to 3.6)	6.99 (6.19 to 7.8)	77.53 (76.22 to 78.85)
JEV2	0.10 (−0.11 to 0.30)	0.87 (0.27 to 1.48)	2.82 (1.74 to 3.90)	0.33 (−0.04 to 0.71)	0.22 (−0.09 to 0.53)	4.38 (3.04 to 5.71)	11.65 (9.56 to 13.74)	79.63 (77.01 to 82.26)
MPSV-A1	0 (0 to 0)	0.01 (−0.02 to 0.04)	4.02 (3.43 to 4.61)	4.20 (3.59 to 4.80)	0.26 (0.10 to 0.41)	2.48 (2.02 to 2.95)	0.28 (0.12 to 0.44)	88.75 (87.80 to 89.7)
MPSV-A2	0.02 (−0.03 to 0.06)	7.04 (6.19 to 7.89)	0.57 (0.32 to 0.82)	0.15 (0.02 to 0.28)	0.01 (−0.02 to 0.05)	7.90 (7.00 to 8.79)	5.45 (4.70 to 6.21)	78.86 (77.50 to 80.21)
MPSV-AC1	0 (0 to 0)	1.37 (0.34 to 2.40)	0.40 (−0.16 to 0.96)	1.59 (0.48 to 2.70)	1.15 (0.20 to 2.10)	1.30 (0.29 to 2.30)	0.06 (−0.16 to 0.27)	94.13 (92.05 to 96.22)
MMR1	0 (0 to 0)	0.11 (0.01 to 0.22)	3.18 (2.63 to 3.74)	26.95 (25.55 to 28.35)	0.87 (0.57 to 1.16)	1.78 (1.37 to 2.20)	57.28 (55.72 to 58.85)	9.82 (8.88 to 10.77)
HepA1	0.18 (0.01 to 0.34)	7.42 (6.38 to 8.46)	3.56 (2.82 to 4.29)	0.28 (0.07 to 0.49)	0.22 (0.04 to 0.41)	3.07 (2.39 to 3.75)	2.41 (1.80 to 3.02)	82.86 (81.37 to 84.35)
Zero^a^	0.17 (0.06 to 0.28)	49.92 (48.57 to 51.27)	0.52 (0.32 to 0.71)	0 (−0.01 to 0.02)	0 (−0.01 to 0.01)	0.30 (0.15 to 0.44)	0.09 (0.01 to 0.17)	49.01 (47.66 to 50.35)
NIP full^b^	0 (0 to 0)	8.54 (7.79 to 9.29)	4.09 (3.56 to 4.62)	0.20 (0.08 to 0.32)	2.68 (2.25 to 3.12)	8.49 (7.74 to 9.24)	0.90 (0.64 to 1.15)	75.11 (73.94 to 76.27)
**Non-NIP vaccines**								
PCV1	0 (0 to 0)	0.34 (0.17 to 0.50)	21.01 (19.88 to 22.15)	24.40 (23.21 to 25.6)	42.93 (41.55 to 44.31)	0.02 (−0.02 to 0.05)	0.02 (−0.02 to 0.05)	11.29 (10.41 to 12.17)
PCV3	0 (0 to 0)	0.68 (0.44 to 0.92)	27.15 (25.84 to 28.46)	22.67 (21.43 to 23.9)	40.94 (39.49 to 42.39)	0.29 (0.13 to 0.45)	0 (−0.01 to 0.02)	8.28 (7.46 to 9.09)
Hib1	0 (0 to 0)	15.02 (14.03 to 16.01)	1.13 (0.83 to 1.42)	0.30 (0.15 to 0.46)	9.57 (8.76 to 10.39)	0.27 (0.13 to 0.42)	1.20 (0.90 to 1.50)	72.51 (71.27 to 73.74)
Hib3	0 (0 to 0)	9.93 (9.04 to 10.82)	3.44 (2.90 to 3.99)	1.14 (0.82 to 1.46)	11.94 (10.97 to 12.91)	0.55 (0.33 to 0.77)	1.52 (1.16 to 1.89)	71.47 (70.12 to 72.82)
Rota1	0 (0 to 0)	10.66 (9.83 to 11.5)	1.16 (0.87 to 1.45)	0.43 (0.26 to 0.61)	15.22 (14.25 to 16.2)	0.10 (0.01 to 0.18)	0.01 (−0.02 to 0.03)	72.41 (71.2 to 73.62)
Rota3	1.08 (0.76 to 1.41)	27.52 (26.11 to 28.92)	1.23 (0.88 to 1.57)	1.94 (1.51 to 2.37)	15.95 (14.8 to 17.1)	0.11 (0.01 to 0.22)	0.37 (0.18 to 0.56)	51.81 (50.24 to 53.38)
Vari1	0.01 (−0.03 to 0.06)	15.52 (14.20 to 16.84)	4.65 (3.88 to 5.42)	1.72 (1.24 to 2.19)	10.30 (9.19 to 11.4)	0.97 (0.61 to 1.32)	0.02 (−0.03 to 0.07)	66.81 (65.1 to 68.53)
EV71 (first)	0 (0 to 0)	7.12 (6.34 to 7.90)	4.05 (3.45 to 4.64)	0.29 (0.13 to 0.46)	15.08 (14.00 to 16.17)	0.18 (0.05 to 0.31)	0.03 (−0.02 to 0.08)	73.25 (71.90 to 74.59)
EV71 (second)	0.34 (0.12 to 0.55)	6.54 (5.63 to 7.45)	0.22 (0.05 to 0.39)	2.32 (1.76 to 2.87)	18.66 (17.22 to 20.1)	0.45 (0.20 to 0.69)	0.18 (0.02 to 0.33)	71.31 (69.64 to 72.98)

Abbreviations: BCG, bacillus Calmette-Guérin vaccine; DTaP, diphtheria and tetanus toxoids and acellular pertussis vaccine; EV71, enterovirus 71 vaccine; hepA, hepatitis A vaccine; hepB, hepatitis B vaccine; Hib, *Haemophilus influenzae* type b vaccine; JEV, Japanese encephalitis vaccine; MMR, measles, mumps, and rubella vaccine; MPSV, meningococcal polysaccharide vaccine; NIP, National Immunization Program; NIP full, fully immunized for age; PCV, pneumococcal conjugate vaccine; PV, poliovirus vaccine; rota, rotavirus vaccine; vari, varicella vaccine; zero, zero dose.

^a^ Zero dose was defined as never having received a single dose of diphtheria-tetanus-pertussis vaccine, PV, or measles, mumps, and rubella vaccine by age 12 months.

^b^ Fully immunized for age was defined as having received all recommended NIP vaccines in the national immunization schedule appropriate for the age of the child observed.

## Discussion

This cross-sectional study analyzed vaccination coverage rates and inequity in coverage for NIP and non-NIP childhood vaccines in China, with 4 primary findings. First, vaccination coverage for each NIP vaccine, stratified by dose, was high, and the proportion of children who were zero dose captured in the survey was 0 in 9 provinces. Despite overall high achievement, some NIP vaccines, like JEV, still have not achieved the 90% target set by the Chinese government to achieve herd immunity and interrupt transmission of vaccine-preventable diseasess.^7,28^ Second, there was a gap between vaccination rates among NIP vaccines and those among non-NIP vaccines. The comparably large contributions of socioeconomic status and place of residence to inequity in non-NIP vaccination coverage suggest that the inclusion of a non-NIP vaccine in the NIP may be associated with substantial reductions in inequality in coverage and increases in the overall mean level of coverage. Third, the issue of equity varied substantially between 2 categories of vaccines in China. All non-NIP vaccines had higher levels of observed inequity in coverage than NIP vaccines. Furthermore, regardless of the level of inequity, the proportion of observed inequity contributed by unfair sources of variation was larger for non-NIP vaccines compared with NIP vaccines. Fourth, decomposition analysis indicated that place of residence and education level of caregivers were important factors associated with coverage for all vaccines, while wealth contributed more to inequalities in non-NIP vaccines.

Through decomposition analyses, it was possible to investigate which factors contributed the most to inequity for each vaccine and dose. For instance, wealth contributed 40.94% (95% CI, 39.49%-42.39%) of the inequity in PCV3, likely associated with high prices in China. For NIP vaccines, unexplained variations contributed the highest proportion of inequities, suggesting that inequality was associated with random noise or unobserved factors, such as personal preferences, vaccines being out of stock, vaccine hesitancy, and missed scheduled appointments. Furthermore, we found that wealth did not make a large contribution to NIP vaccine coverage, contrary to findings from previous studies in China.^29^ This suggests that using a composite equity metric may allow researchers to disentangle inequities that may be masked by or wrongly associated with socioeconomic status alone. Among non-NIP vaccines, substantial proportions of unexplained variation in observed inequalities still existed for all non-NIP vaccines except PCV. This suggests that the price of most non-NIP vaccines may not be the largest obstacle to coverage, particularly in the context of China’s rapidly growing gross domestic product per capita.^30^ Unexplained variations among non-NIP vaccines may be associated with similar unobserved factors as those for NIP vaccines. For most non-NIP vaccines, important barriers to increasing vaccination coverage rates may include less widely disseminated government recommendations for routine immunization policy, as well as a lack of widespread availability of these vaccines across provinces and facilities. Additionally, we observed that caregiver education made a large contribution to inequalities in coverage for all vaccines. Caregiver education, particularly that of mothers, has been found to be an important factor associated with child health globally.^31,32^ Our results suggest that policies targeting improved education, in particular maternal education, may also be associated with increases in child vaccination coverage rates and decreases in vaccination inequality.^33,34^

Other studies have examined inequity over a single dimension, were typically based on socioeconomic status, or decomposed wealth-based inequity into multiple dimensions. By contrast, the VERSE model ranking procedure accounted for multidimensional inequity and then parsed out relative contributions of factors to overall inequity in vaccination status. Such data may be valuable for health policy makers in diagnosing problems in inequity and coverage and better targeting interventions to address factors with the largest contributions. For example, health insurance coverage or a conditional cash-transfer program may be less likely to work in a setting where socioeconomic status was not a substantial contributor to observed inequity. Additionally, concentration index values for NIP vaccines in this study were smaller than in 2 studies^29,35^ that used monthly household income as the ranking reference to calculate concentration index values for vaccination in China. This may be due to the association of socioeconomic status with fair sources of variation in outcomes, such as the age of the child in a particular birth cohort, which were explicitly accounted for in the VERSE model.

### Limitations

This study has several limitations. First, recall bias, such as for monthly incomes, may occur in self-reported responses, leading to mismeasurement of sociodemographic factors. Second, factors included in this study may not be comprehensive for fully ranking individuals by unfair disadvantage or may not be able to explain potentially important factors associated with inequality owing to insufficient data availability. Third, owing to limitations on sample size, the survey was representative at the national level but possibly not at the provincial level. Therefore, resulting subnational estimates and interpretations were internally valid for only the population surveyed, rather than being representative of the provincial level.^18^

## Conclusions

This cross-sectional study’s findings suggest that the routine childhood immunization policy in China made great achievements, with coverage rates of all NIP vaccines remaining at a high level. For non-NIP vaccines, coverage rates were comparatively low and larger amounts of inequity existed. These findings suggest that interventions should consider not only the price of vaccines or household incomes as systemic barriers, but also education level and health awareness of primary caregivers and vaccine supply distribution across geographic settings. Our findings additionally suggest that inclusion within the NIP may be an important strategy for improving non-NIP vaccine coverage rates and reducing inequities in vaccination status.

## Notes

Supplement 1. **eMethods 1.** Sample Size Calculation in Survey **eMethods 2.** Details of Vaccine Economics Research for Sustainability and Equity Model **eFigure 1.** Provinces and Municipalities Selected for Study **eFigure 2.** Flow Chart of Location Selection and Participant Recruitment **eTable 1.** Demographic Characteristics of Children With vs Without Complete Vaccination Records **eTable 2.** Standard Schedules of National Immunization Program Vaccines **eTable 3.** Standard Schedules of Non–National Immunization Program Vaccines **eTable 4.** Absolute Equity Gap by Vaccine and Dose **eFigure 3.** Coverage-Equity Trade-off Plane for Vaccines **eFigure 4.** Decomposition of Vaccine Inequity

Supplement 2.
Data Sharing Statement
