# Supplementary material for: Coverage and Equity of Childhood Vaccines in China
Source: JAMA Netw Open. 2022 Dec 9;5(12):e2246005. doi: 10.1001/jamanetworkopen.2022.46005 (PMC9856225; doi:10.1001/jamanetworkopen.2022.46005)
Supplement: Supplement 1. — eMethods 1. Sample Size Calculation in Survey eMethods 2. Details of Vaccine Economics Research for Sustainability and Equity Model eFigure 1. Provinces and Municipalities Selected for Study eFigure 2. Flow Chart of Location Selection and Participant Recruitment eTable 1. Demographic Characteristics of Children With vs Without Complete Vaccination Records eTable 2. Standard Schedules of National Immunization Program Vaccines eTable 3. Standard Schedules of Non–National Immunization Program Vaccines eTable 4. Absolute Equity Gap by Vaccine and Dose eFigure 3. Coverage-Equity Trade-off Plane for Vaccines eFigure 4. Decomposition of Vaccine Inequity [file jamanetwopen-e2246005-s001.pdf]

Supplemental Online Content

Zhang H, Lai X, Mak J, et al. Coverage and equity of childhood vaccines in China. *JAMA Netw Open*. 2022;5(12):e2246005. doi:10.1001/jamanetworkopen.2022.46005

eMethods 1. Sample Size Calculation in Survey
eMethods 2. Details of Vaccine Economics Research for Sustainability and Equity Model
eFigure 1. Provinces and Municipalities Selected for Study
eFigure 2. Flow Chart of Location Selection and Participant Recruitment
eTable 1. Demographic Characteristics of Children With vs Without Complete Vaccination Records
eTable 2. Standard Schedules of National Immunization Program Vaccines
eTable 3. Standard Schedules of Non–National Immunization Program Vaccines
eTable 4. Absolute Equity Gap by Vaccine and Dose
eFigure 3. Coverage-Equity Trade-off Plane for Vaccines
eFigure 4. Decomposition of Vaccine Inequity

This supplemental material has been provided by the authors to give readers additional information about their work.

© 2022 Zhang H et al. *JAMA Network Open.*

eMethods 1. Sample Size Calculation in Survey

This study applied the household-based cluster survey method recommended by the WHO.[1] The sample size of this survey was calculated based on the formula as follows:

$$n = \frac{Z_{1-\alpha/2}^{2} \cdot P \cdot (1-P)}{\delta^{2}}$$

where δ is the permissible error, a two-tailed α error is 5%, confidence level 1-α is the degree of confidence, and P is the vaccination rate of 6-59 month-old children. In this study, the confidence level of 95% is taken, and the error does not exceed 5%. So $Z^2_{(1-\alpha/2)}$ is equal to 1.96. According to the calculation formula, the closer the vaccination rate P is to 50%, the larger the required sample size (the most conservative estimated sample size). At this time, the minimum valid sample size is n=((1.96)^2·0.5·(1-0.5))/(0.05)^2 ≈384. The smallest sample size of each province is 384 children, and the smallest sample size of children surveyed would be 3840 for ten provinces totally. In practice, we collected a larger sample size than expected to increase the reliability of the results.

eReference:

1. Cutts FT. The use of the WHO cluster survey method for evaluating the impact of the expanded programme on immunization on target disease incidence. The Journal of tropical medicine and hygiene. 1988;91:231-9.

© 2022 Zhang H et al. *JAMA Network Open.*

eMethods 2. Details of Vaccine Economics Research for Sustainability and Equity Model

The composite vaccination equity assessment metric in the Vaccine Economics Research for Sustainability & Equity (VERSE) model is derived from literature on the measurement of socioeconomic equity by Wagstaff and Erreygers combined with measures of direct unfairness in healthcare access outlined in the works of Fleurbaey, Schokkaert, Cookson, and Barbosa [1–8]. The composite metric takes the form of a concentration index of vaccination coverage, where instead of ranking individuals by income, individuals are ranked by a multi-dimensional score of unfair disadvantage in access. Unfair disadvantage as parameterized in the VERSE metric is an adaptation of a direct unfairness measure. It computes the predicted vaccination coverage from a logistic model-based, for binary outcomes, or a generalized linear model, for continuous outcomes, upon multiple dimensions of fair and unfair sources of variation in vaccination coverage [5].

Fair sources of variation in coverage include whether the child is underage to receive the vaccine according to Chinese national immunization schedule for NIP vaccines and expert consensus for non-NIP vaccines. We used the birth date and the survey date to calculate the age of a child and compared it with the appropriate vaccination schedule to determine whether the child was underage. Unfair sources of variation included in the standard model are the gender of the child, and respondents (caregivers)' education level, socioeconomic status (monthly family income per capita), medical insurance, place of residence (urban or rural), and provinces. These were chosen based on stakeholder engagement and near-universal data collection on these dimensions through national health information systems [9]. The direct unfairness ranking metric is then assessed as the predicted probability of vaccination, holding the fair determinants at reference levels and allowing the unfair determinants to vary. For continuous variables, the predicted value of the continuous output holds the fair determinants at reference levels and allows the unfair determinants to vary. This unfair disadvantage metric is then utilized as the ranking variable in a concentration index, alongside the cumulative share of the outcome, to compute the composite coverage equity metric.

For the binary case of vaccination coverage where the outcome takes a value of 1 if the child receives the vaccine and 0 otherwise, the direct unfairness metric for vaccination coverage indicator (vci_{du}) can be written as:

$$vci_{du} = vci_{predicted}\,(\boldsymbol{N}_{ref}, \boldsymbol{P}_{ref}, \boldsymbol{Z}_{i}, \boldsymbol{X}_{ref})$$

where:

$\boldsymbol{N}$ = Vector of need variables (in the vaccine case, only the age of the child is used)

© 2022 Zhang H et al. *JAMA Network Open.*

$\boldsymbol{P}$ = Vector of preference for healthcare variables

$\boldsymbol{Z}$ = Vector of unfair variables (e.g., socioeconomic status, urban/rural, sex of the child, caregiver's education)

$\boldsymbol{X}$ = Vector of neither fair nor unfair variables (e.g., variables that may confound the relationship between unfair predictors and coverage)

$vci_{predicted}$ = Predicted probability of receiving vaccines holding need (N) & neutral (X) variables at reference levels

For the VERSE model, the basic assumption is that there are no neutral variables and that parental (or caregiver's) preferences for vaccination are either not observable or are a function of the $\boldsymbol{Z}$ vector variables (e.g., parental education) and therefore should be counted as unfair sources of variation and not true preferences. As such, the direct unfairness in vaccination coverage indicator (vci_{du}) can be simplified as:

$$vci_{du} = vci_{predicted} \, (\boldsymbol{N}_{ref}, \boldsymbol{Z}_i)$$

Therefore, under the logistic framework letting vaccination status (v) = 1 if vaccinated and 0 otherwise, the predicted vci_{du} can be written as:

Let $p_i = \Pr(v = 1 | \, \boldsymbol{N}_{ref}, \boldsymbol{Z}_i)$

$\text{Logit}(p_i) = \alpha + \beta Z_i + \gamma \boldsymbol{N}_{ref} + \varepsilon_i$

Using this setup, the predicted values are defined by: $\widehat{p_i} = vci_{du}$.

Once vci_{du} is obtained, it is then used as the ranking variable to compute a Wagstaff's concentration index, replacing the more traditional ranking variable of socioeconomic status [6,8]. As such, the predicted probability of vaccination conditional on unfair determinants (vci_{du}), or in the continuous case the predicted healthcare access level based on unfair determinants, functions in the same manner as a wealth index creating a scale where the relative rank of individuals over (vci_{du}) depicts their degree of relative unfair disadvantage in obtaining the outcome in question. This is utilized alongside the cumulative share of attainment of the health outcome to compute the final index, which, as a concentration-style index, exhibits the properties of a Gini-index: bounded between -1 and 1 and, therefore, standardized by construction.

The VERSE toolkit enables the production of a traditional Wagstaff concentration index (CI_W):

$$CI_W = \frac{2\, Cov(vci_{direct}, F(vci_{du}))}{\mu vc}$$

Where:

vci_{direct} = Directly standardized individual level of healthcare (observed vaccination coverage)

$F(vci_{du})$ = The cumulative distribution function of direct unfairness

μvc = Mean level of healthcare (vaccination coverage) across the entire population

Since the metric is based Wagstaff's concentration index, regression-based Kitagawa-Blinder-Oaxaca decomposition can be employed to generate the cumulative share of overall observed inequality relating to each of the fair and unfair predictor[3,10–11].

Finally, it is possible to compute the Absolute Equity Gap derived from the concentration index above. This involves subtracting the outcome from the top 20% of the study sample (ranked by multidimensional unfairness) and the bottom 20% of the study sample.

$$AEG = hci_{observed}(top\ 20\%\ (F(hci_{du}))) - hci_{observed}(Bottom\ 20\%\ (F(hci_{du})))$$

A fundamental assumption of the VERSE model is that every child should be vaccinated by the recommended age in the national immunization schedule. As such, the only source of fair variation in vaccination status should be the *age of the child*. This means that children who are younger than the recommended age for a specific vaccination can fairly be expected not to have received a vaccination and should be netted out of the unfair disadvantage metric computation process. All other sources of variation in vaccination status (socioeconomic status, gender of the child, and respondents (caregivers)' education level, socioeconomic status (monthly family income per capita), medical insurance, place of residence (urban or rural), and province) should be considered as unfair sources of vaccination coverage. The reference levels for all determinants in the analysis are set at the subnational level, so negative indices will signal a protective relationship between unfair risk factors and outcomes. Such negative values will indicate a pro-disadvantaged distribution of vaccination within that sub-unit with respect to national-level drivers of disadvantage.

eReferences:

1. Patenaude B, Odihi D, Sriudomporn S, Mak J, Watts E, de Broucker G. A standardized approach for measuring multivariate equity in vaccination coverage, cost-of-illness, and health outcomes: Evidence from the Vaccine Economics Research for Sustainability & Equity (VERSE) project. Social science & medicine. 2022;302:114979.

© 2022 Zhang H et al. *JAMA Network Open.*

2. Alonge O, Peters DH. Utility and limitations of measures of health inequities: a theoretical perspective. *Global Health Action* 2015; **8**: 27591.
3. Barbosa EC, Cookson R. Multiple inequity in health care: An example from Brazil. *Social Science & Medicine* 2019; **228**: 1–8.
4. Williams AH, Cookson RA. Equity–efficiency trade-offs in health technology assessment. *Int J Technol Assess Health Care* 2006; **22**: 1–9.
5. Fleurbaey M, Schokkaert E. Chapter Sixteen - Equity in Health and Health Care. *Handbook of health economics* 2011; **2**: 1003–92.
6. Wagstaff A. The concentration index of a binary outcome revisited. *Health Econ* 2011; **20**: 1155–60.
7. Fleurbaey M, Schokkaert E. Unfair inequalities in health and health care. *Journal of Health Economics* 2009; **28**: 73–90.
8. O'Donnell O, van Doorslaer E, Wagstaff A, Lindelow M. Analyzing Health Equity Using Household Survey Data. World Bank Publications, 2007.
9. DHS. The DHS Program - Quality Information To Plan, Monitor And Improve Population, Health, And Nutrition Programs. DHS. https://dhsprogram.com/ (accessed Oct 12, 2021).
10. Blinder AS. Wage Discrimination: Reduced Form and Structural Estimates. *The Journal of Human Resources* 1973; **8**: 436.
11. Oaxaca R. Male-Female Wage Differentials in Urban Labor Markets. *International Economic Review* 1973; **14**: 693.

© 2022 Zhang H et al. *JAMA Network Open.*

eFigure 1. Provinces and Municipalities Selected for Study

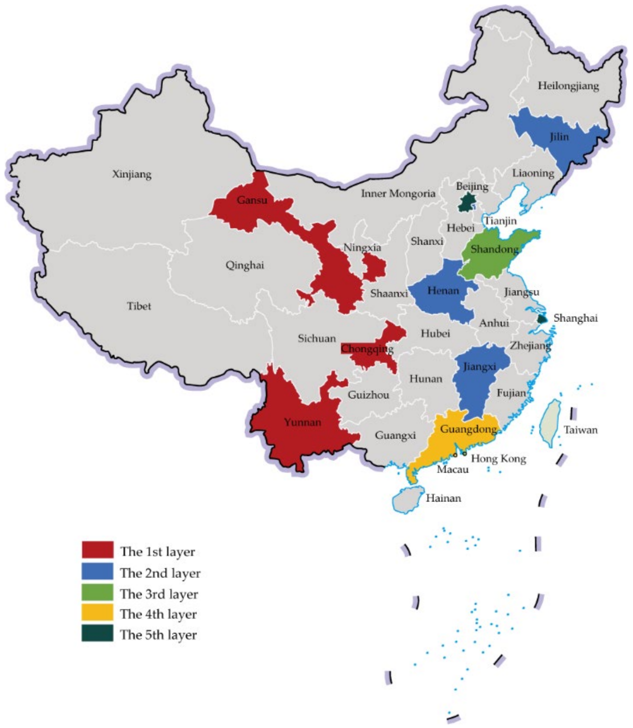

Note: The 1st layer includes the least developed provinces, and the 5th layer includes the most developed provinces in China. The ten provinces/municipalities (equivalent to provinces) selected in the mainland China were Chongqing, Gansu, and Yunnan (the 1st layer); Henan, Jiangxi, and Jilin (the 2nd layer); Shandong (the 3rd layer); Guangdong (the 4th layer); and Beijing and Shanghai (the 5th layer). There were more provinces/municipalities in the first and second layers, so three provinces/municipalities were selected for each layer.

© 2022 Zhang H et al. *JAMA Network Open.*

eFigure 2. Flow Chart of Location Selection and Participant Recruitment

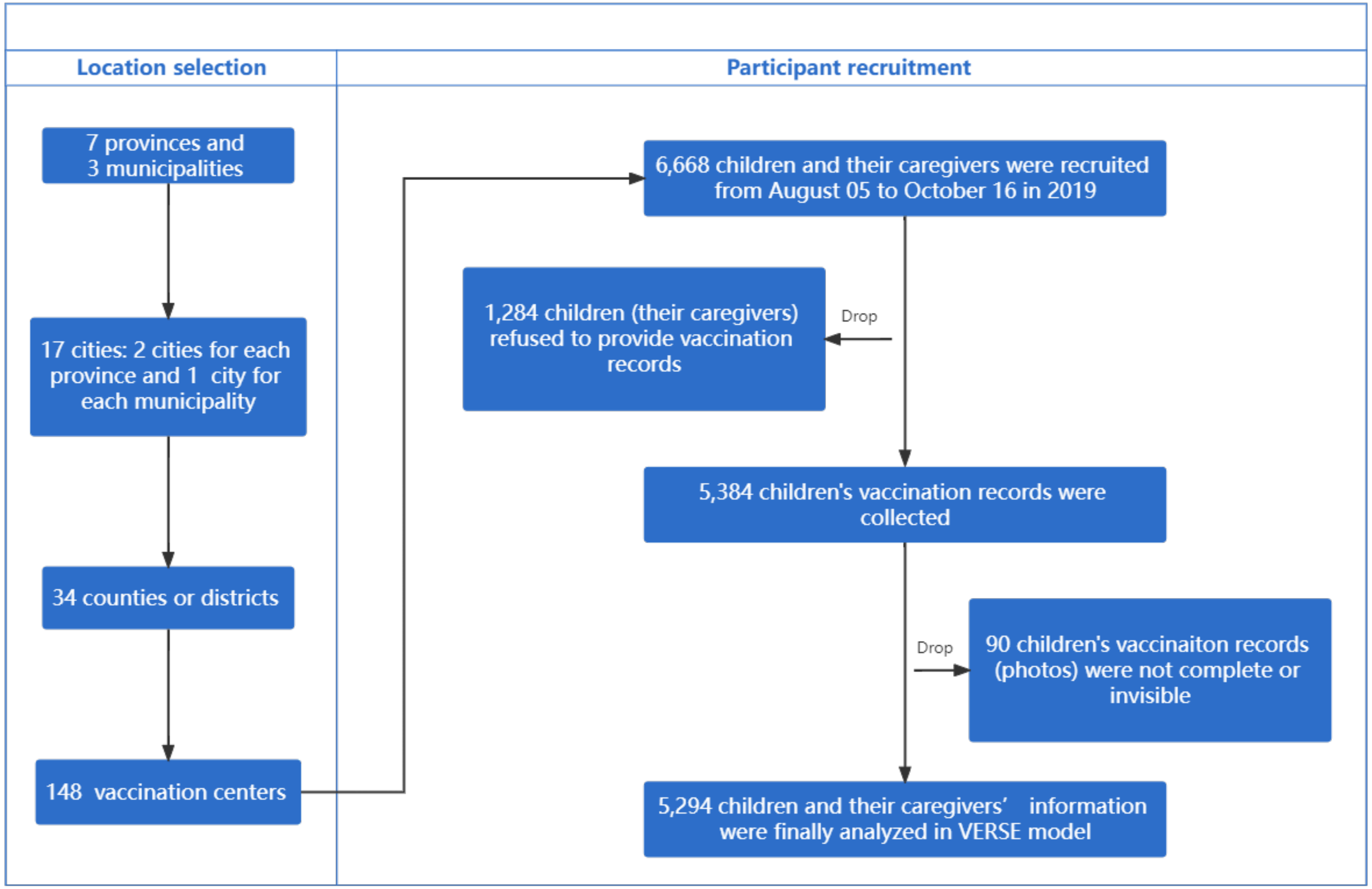

© 2022 Zhang H et al. *JAMA Network Open.*

eTable 1. Demographic Characteristics of Children With vs Without Complete Vaccination Records

	Children with complete vaccination records (%)	Children without vaccination records (%)
National level characteristics		
	n=5294	n=1374
Age (months)		
6-11	1439 (27.2)	329 (23.9)
12-23	1547 (29.2)	412 (30.0)
24-35	981 (18.5)	260 (18.9)
36-59	1327 (25.1)	373 (27.2)
Gender		
Male	2796 (52.8)	701 (51.0)
Female	2498 (47.2)	673 (49.0)
Age of the respondent (years)		
< 30	1637 (30.9)	412 (30.0)

© 2022 Zhang H et al. *JAMA Network Open.*

30-39	2457 (46.4)	582 (42.4)
40-49	458(8.7)	111 (8.1)
≥50	742(14.0)	269 (19.5)
Family relationship between respondent and child		
Mother	3534(66.8)	871(63.4)
Father	907(17.1)	215(15.1)
Grandparent	853(16.1)	288(21.5)
Education level [a]		
Elementary school and below	521(9.8)	153(11.1)
Junior high school	1355(25.6)	355(25.8)
High school/Vocational school	1194(22.6)	309(22.5)
Associate degree (2 years)	997(18.8)	253(18.4)
Bachelor degree and above	1227(23.2)	304(22.1)
Place of residence		

© 2022 Zhang H et al. *JAMA Network Open.*

Rural	2139(40.4)	675(49.1)
Urban	3155(59.6)	699(50.9)
Quantiles of per capita monthly income [b]		
Quantile 1 (CNY < 1,000)	1152(21.8)	393 (28.6)
Quantile 2 (CNY 1,001-1,600)	900(17.0)	246 (17.9)
Quantile 3 (CNY 1,601-2,400)	1063(20.1)	274 (20.0)
Quantile 4 (CNY 2,401-3,750)	1132(21.4)	268 (19.5)
Quantile 5 (CNY >3,751)	1047(19.8)	193 (14.0)
Medical insurance type [a]		
Urban rural resident medical insurance	3234(61.1)	847 (61.7)
Urban employee based medical insurance	1885(35.6)	495 (36.0)
No medical insurance	175(3.3)	32 (2.3)

Note: a, The education level and medical insurance type refer to those of the adult respondents;

b, CNY 1= USD 0.145 in 2019.

© 2022 Zhang H et al. *JAMA Network Open.*

eTable 2. Standard Schedules of National Immunization Program Vaccines

				Age														
**Vaccine name**	**Vaccination approaches**	**Dose**	**English abbreviations**	**birth**	**1 month**	**2 month**	**3 month**	**4 month**	**5 month**	**6 month**	**8 month**	**9 month**	**18 month**	**2 yr**	**3 yr**	**4 yr**	**5 yr**	**6 yr**
**Hepatitis B vaccine**	**Intramuscular injection**	**10 or 20ug**	**HepB**	**1**	**2**					**3**								
**Bacillus calmette-guérin vaccine**	**Intramuscular injection**	**0.1ml**	**BCG**	**1**														
**Polio vaccine**	**Intramuscular injection**	**0.5ml**	**IPV**			**1**	**2**											
	**Oral**	**A pill**	**OPV**					**3**								**4**		
**Diphtheria and tetanus toxoids and acellular pertussis vaccine**	**Intramuscular injection**	**0.5ml**	**DTaP**				**1**	**2**	**3**				**4**					
	**Intramuscular injection**	**0.5ml**	**DT**															**5**
**Measles, mumps, and rubella vaccine**	**Subcutaneous injection**	**0.5ml**	**MMR**								**1**		**2**					
**Japanese encephalitis vaccine**	**Subcutaneous injection**	**0.5ml**	**JE-L**								**1**			**2**				
	**Intramuscular injection**	**0.5ml**	**JE-I**								**1、2**				**3**			**4**
**Meningococcal polysaccharide vaccine**	**Subcutaneous injection**	**0.5ml**	**MPSV-A**							**1**		**2**						
	**Subcutaneous injection**	**0.5ml**	**MPSV-AC**												**3**			**4**
**Hepatitis A vaccine**	**Subcutaneous injection**	**0.5ml**	**HepA-L**										**1**					
	**Intramuscular injection**	**0.5 or 0.1ml**	**HepA-I**										**1**	**2**				

© 2022 Zhang H et al. *JAMA Network Open.*

eTable 3. Standard Schedules of Non–National Immunization Program Vaccines

<table>
<tr><th></th><th></th><th></th><th colspan="14">Age</th></tr>
<tr><th>Vaccine name</th><th>Vaccination approaches</th><th>English abbreviations</th><th>1.5 month</th><th>2 month</th><th>3 month</th><th>4 month</th><th>5 month</th><th>6 month</th><th>8 month</th><th>12 month</th><th>18 month</th><th>2 yr</th><th>3 yr</th><th>4 yr</th><th>5 yr</th><th>6 yr</th></tr>
<tr><td>Pneumococcal conjugate vaccine</td><td>Intramuscular injection</td><td>PCV</td><td colspan="6">3 doses of primary immunization, intervals are 4-8 weeks</td><td></td><td>4</td><td></td><td></td><td></td><td></td><td></td><td></td></tr>
<tr><td>Haemophilus influenzae type b vaccine;</td><td>Intramuscular injection</td><td>Hib</td><td></td><td>1</td><td>2</td><td>3</td><td></td><td></td><td></td><td colspan="2">4</td><td></td><td></td><td></td><td></td><td></td></tr>
<tr><td>Rotavirus vaccine</td><td>Oral</td><td>Rota</td><td></td><td colspan="10">one dose per year</td><td></td><td></td><td></td></tr>
<tr><td>Varicella vaccine</td><td>Intramuscular injection</td><td>Vari</td><td></td><td></td><td></td><td></td><td></td><td></td><td></td><td colspan="2">1</td><td></td><td></td><td>2</td><td></td><td></td></tr>
<tr><td>Enterovirus 71 vaccine</td><td>Intramuscular injection</td><td>EV71</td><td></td><td></td><td></td><td></td><td></td><td colspan="8">2 doses, interval is one month</td><td></td></tr>
</table>

Note: The immunization schedules of non-National Immunization Program vaccines are different by province in China, so the present study follows childhood immunization program in Shanghai as the standard schedule.

© 2022 Zhang H et al. *JAMA Network Open.*

eTable 4. Absolute Equity Gap by Vaccine and Dose

Vaccines	Province (95% CI)										National (95% CI)
	Beijing	Chongqing	Gansu	Guangdong	Henan	Jiangxi	Jilin	Shandong	Shanghai	Yunnan	
**NIP vaccines**											
BCG	0.015	-0.008	0.000	0.036	0.009	0.009	0.000	0.009	0.026	0.028	0.015
	(0.007 to 0.023)	(-0.016 to 0.000)	(-0.006 to 0.006)	(0.028 to 0.044)	(0.003 to 0.015)	(0.003 to 0.015)	(-0.008 to 0.008)	(0.001 to 0.017)	(0.020 to 0.032)	(0.020 to 0.036)	(0.007 to 0.023)
DTaP1	-0.009	0.000	0.041	0.051	0.010	0.018	-0.008	0.000	0.000	0.019	0.021
	(-0.017 to -0.001)	(-0.008 to 0.008)	(0.033 to 0.049)	(0.043 to 0.059)	(0.002 to 0.018)	(0.010 to 0.026)	(-0.016 to 0.000)	(-0.008 to 0.008)	(-0.008 to 0.008)	(0.011 to 0.027)	(0.011 to 0.031)
DTaP3	0.022	0.022	0.089	0.107	0.016	0.088	0.000	0.074	-0.001	0.039	0.079
	(0.004 to 0.040)	(0.002 to 0.042)	(0.069 to 0.109)	(0.087 to 0.127)	(-0.006 to 0.038)	(0.068 to 0.108)	(-0.020 to 0.020)	(0.054 to 0.094)	(-0.021 to 0.019)	(0.021 to 0.057)	(0.057 to 0.101)
PV1	0.009	0.000	0.000	0.019	0.019	0.008	0.000	0.000	0.000	0.000	0.008
	(0.005 to 0.013)	(-0.004 to 0.004)	(-0.002 to 0.002)	(0.017 to 0.021)	(0.015 to 0.023)	(0.004 to 0.012)	(-0.004 to 0.004)	(-0.004 to 0.004)	(-0.004 to 0.004)	(-0.004 to 0.004)	(0.002 to 0.014)
PV2	0.015	0.040	0.043	0.010	0.038	0.019	0.000	0.043	0.021	0.020	0.03
	(0.007 to 0.023)	(0.030 to 0.05)	(0.035 to 0.051)	(0.002 to 0.018)	(0.030 to 0.046)	(0.011 to 0.027)	(-0.008 to 0.008)	(0.035 to 0.051)	(0.013 to 0.029)	(0.012 to 0.028)	(0.018 to 0.042)
PV3	0.043	0.066	0.047	0.022	0.055	0.049	-0.009	0.078	0.012	0.024	0.052
	(0.029 to 0.057)	(0.052 to 0.08)	(0.033 to 0.061)	(0.006 to 0.038)	(0.039 to 0.071)	(0.033 to 0.065)	(-0.023 to 0.005)	(0.066 to 0.09)	(-0.002 to 0.026)	(0.010 to 0.038)	(0.036 to 0.068)

© 2022 Zhang H et al. *JAMA Network Open.*

HepB1	0.000	0.000	0.000	0.020	0.020	0.009	0.008	0.009	0.019	0.009		0.017
	(-0.006 to 0.006)	(-0.008 to 0.008)	(-0.006 to 0.006)	(0.014 to 0.026)	(0.014 to 0.026)	(0.003 to 0.015)	(0.002 to 0.014)	(0.003 to 0.015)	(0.013 to 0.025)	(0.005 to 0.013)		(0.009 to 0.025)
HepB3	0.012	0.000	0.000	0.046	0.012	-0.001	0.042	0.025	0.012	0.033		0.030
	(0.000 to 0.024)	(-0.012 to 0.012)	(-0.012 to 0.012)	(0.034 to 0.058)	(0.000 to 0.024)	(-0.013 to 0.011)	(0.030 to 0.054)	(0.013 to 0.037)	(0.000 to 0.024)	(0.021 to 0.045)		(0.018 to 0.042)
JEV1	0.095	-0.001	0.070	0.035	0.040	0.037	0.009	0.039	0.011	0.021		0.057
	(0.079 to 0.111)	(-0.019 to 0.017)	(0.054 to 0.086)	(0.019 to 0.051)	(0.024 to 0.056)	(0.019 to 0.055)	(-0.009 to 0.027)	(0.023 to 0.055)	(-0.005 to 0.027)	(0.005 to 0.037)		(0.037 to 0.077)
JEV2	0.120	-0.043	0.000	0.047	0.316	0.200	0.105	0.105	0.036	0.137		0.193
	(0.063 to 0.177)	(-0.112 to 0.026)	(-0.069 to 0.069)	(-0.020 to 0.114)	(0.251 to 0.381)	(0.135 to 0.265)	(0.036 to 0.174)	(0.042 to 0.168)	(-0.031 to 0.103)	(0.074 to 0.200)		(0.122 to 0.264)
MPSV-A1	0.017	0.044	0.049	0.075	0.066	0.033	-0.009	0.021	0.006	0.031		0.047
	(0.001 to 0.033)	(0.028 to 0.06)	(0.033 to 0.065)	(0.057 to 0.093)	(0.050 to 0.082)	(0.015 to 0.051)	(-0.027 to 0.009)	(0.005 to 0.037)	(-0.008 to 0.02)	(0.015 to 0.047)		(0.029 to 0.065)
MPSV-A2	0.064	0.072	0.021	0.028	0.047	0.127	0.013	0.137	0.002	0.023		0.136
	(0.037 to 0.091)	(0.043 to 0.101)	(-0.006 to 0.048)	(-0.003 to 0.059)	(0.018 to 0.076)	(0.098 to 0.156)	(-0.016 to 0.042)	(0.108 to 0.166)	(-0.025 to 0.029)	(-0.004 to 0.050)		(0.107 to 0.165)
MPSV-AC1	0.250	0.125	0.200	0.083	0.000	0.222	0.000	0.133	0.202	0.200		0.190
	(0.193 to 0.307)	(0.060 to 0.190)	(0.143 to 0.257)	(0.014 to 0.152)	(-0.073 to 0.073)	(0.163 to 0.281)	(-0.057 to 0.057)	(0.066 to 0.200)	(0.145 to 0.259)	(0.135 to 0.265)		(0.114 to 0.266)
MMR1	0.012	0.011	0.036	0.052	0.025	0.038	0.043	0.000	0.000	0.011		0.037
	(0.000 to 0.024)	(-0.001 to 0.023)	(0.024 to 0.048)	(0.042 to 0.062)	(0.013 to 0.037)	(0.028 to 0.048)	(0.031 to 0.055)	(-0.010 to 0.010)	(-0.012 to 0.012)	(-0.001 to 0.023)		(0.023 to 0.051)

© 2022 Zhang H et al. *JAMA Network Open.*

HepA1	-0.027	0.001	0.025	0.107	0.063	-0.052	0.24	0.04	-0.038	0.101		0.096
	(-0.058 to 0.004)	(-0.032 to 0.034)	(-0.008 to 0.058)	(0.076 to 0.138)	(0.032 to 0.094)	(-0.083 to -0.021)	(0.207 to 0.273)	(0.009 to 0.071)	(-0.067 to -0.009)	(0.07 to 0.132)		(0.065 to 0.127)
ZERO	0.000	0.000	0.000	0.000	0.000	0.000	0.000	0.000	0.000	0.002		0.000
	(0.000 to 0.000)	(0.000 to 0.000)	(0.000 to 0.000)	(0.000 to 0.000)	(0.000 to 0.000)	(0.000 to 0.000)	(0.000 to 0.000)	(0.000 to 0.000)	(0.000 to 0.000)	(0.002 to 0.002)		(0.000 to 0.000)
NIP FULL	-0.021	0.092	0.138	0.050	0.066	0.143	0.018	-0.003	0.041	0.078		0.111
	(-0.050 to 0.008)	(0.061 to 0.123)	(0.107 to 0.169)	(0.019 to 0.081)	(0.035 to 0.097)	(0.112 to 0.174)	(-0.013 to 0.049)	(-0.034 to 0.028)	(0.010 to 0.072)	(0.047 to 0.109)		(0.08 to 0.142)
**Non-NIP vaccines**												
PCV1	0.262	0.192	0.155	0.206	0.092	0.135	0.017	0.058	0.235	0.185		0.231
	(0.238 to 0.286)	(0.168 to 0.216)	(0.131 to 0.179)	(0.184 to 0.228)	(0.068 to 0.116)	(0.111 to 0.159)	(-0.005 to 0.039)	(0.034 to 0.082)	(0.213 to 0.257)	(0.163 to 0.207)		(0.204 to 0.258)
PCV3	0.193	0.128	0.109	0.148	0.033	0.072	0.018	0.033	0.201	0.099		0.165
	(0.171 to 0.215)	(0.108 to 0.148)	(0.089 to 0.129)	(0.126 to 0.17)	(0.013 to 0.053)	(0.050 to 0.094)	(-0.002 to 0.038)	(0.013 to 0.053)	(0.181 to 0.221)	(0.079 to 0.119)		(0.14 to 0.19)
Hib1	0.208	0.342	0.614	0.278	0.120	-0.199	0.040	-0.067	0.229	0.376		0.603
	(0.167 to 0.249)	(0.299 to 0.385)	(0.573 to 0.655)	(0.235 to 0.321)	(0.077 to 0.163)	(-0.242 to -0.156)	(-0.003 to 0.083)	(-0.11 to -0.024)	(0.190 to 0.268)	(0.333 to 0.419)		(0.570 to 0.636)
Hib3	0.106	0.391	0.266	0.371	0.302	-0.14	0.019	-0.095	0.341	0.316		0.531
	(0.065 to 0.147)	(0.352 to 0.43)	(0.227 to 0.305)	(0.332 to 0.410)	(0.263 to 0.341)	(-0.179 to -0.101)	(-0.022 to 0.060)	(-0.134 to -0.056)	(0.302 to 0.380)	(0.277 to 0.355)		(0.498 to 0.564)
Rota1	0.119	0.364	0.278	0.034	0.161	-0.191	0.025	-0.152	0.195	0.305		0.378
	(0.086 to 0.152)	(0.329 to 0.399)	(0.245 to 0.311)	(-0.001 to 0.069)	(0.128 to 0.194)	(-0.224 to -0.158)	(-0.008 to 0.058)	(-0.185 to -0.119)	(0.164 to 0.226)	(0.272 to 0.338)		(0.347 to 0.409)
Rota3	0.000	0.022	0.000	0.023	0.000	-0.013	0.000	0.000	0.132	0.057		0.060

© 2022 Zhang H et al. *JAMA Network Open.*

	(-0.010 to 0.010)	(0.012 to 0.032)	(-0.012 to 0.012)	(0.011 to 0.035)	(-0.012 to 0.012)	(-0.025 to -0.001)	(-0.012 to 0.012)	(-0.01 to 0.01)	(0.122 to 0.142)	(0.043 to 0.071)		(0.044 to 0.076)
Vari1	0.155	0.244	0.550	0.083	0.085	-0.14	-0.129	0.040	-0.010	0.140		0.434
	(0.102 to 0.208)	(0.191 to 0.297)	(0.497 to 0.603)	(0.030 to 0.136)	(0.032 to 0.138)	(-0.193 to -0.087)	(-0.182 to -0.076)	(-0.013 to 0.093)	(-0.063 to 0.043)	(0.087 to 0.193)		(0.385 to 0.483)
EV71 (1st)	-0.025	0.305	0.469	0.185	0.107	-0.133	0.088	-0.151	0.151	0.409		0.382
	(-0.070 to 0.020)	(0.260 to 0.350)	(0.424 to 0.514)	(0.140 to 0.230)	(0.062 to 0.152)	(-0.178 to -0.088)	(0.041 to 0.135)	(-0.196 to -0.106)	(0.106 to 0.196)	(0.364 to 0.454)		(0.339 to 0.425)
EV71 (2nd)	-0.085	0.399	0.292	0.211	0.31	-0.012	0.048	-0.042	0.147	0.355		0.357
	(-0.132 to -0.038)	(0.348 to 0.450)	(0.241 to 0.343)	(0.160 to 0.262)	(0.259 to 0.361)	(-0.061 to 0.037)	(-0.003 to 0.099)	(-0.093 to 0.009)	(0.098 to 0.196)	(0.308 to 0.402)		(0.310 to 0.404)

Abbreviations: AEG = Absolute Equity Gap.

© 2022 Zhang H et al. *JAMA Network Open.*

eFigure 3. Coverage-Equity Trade-off Plane for Vaccines

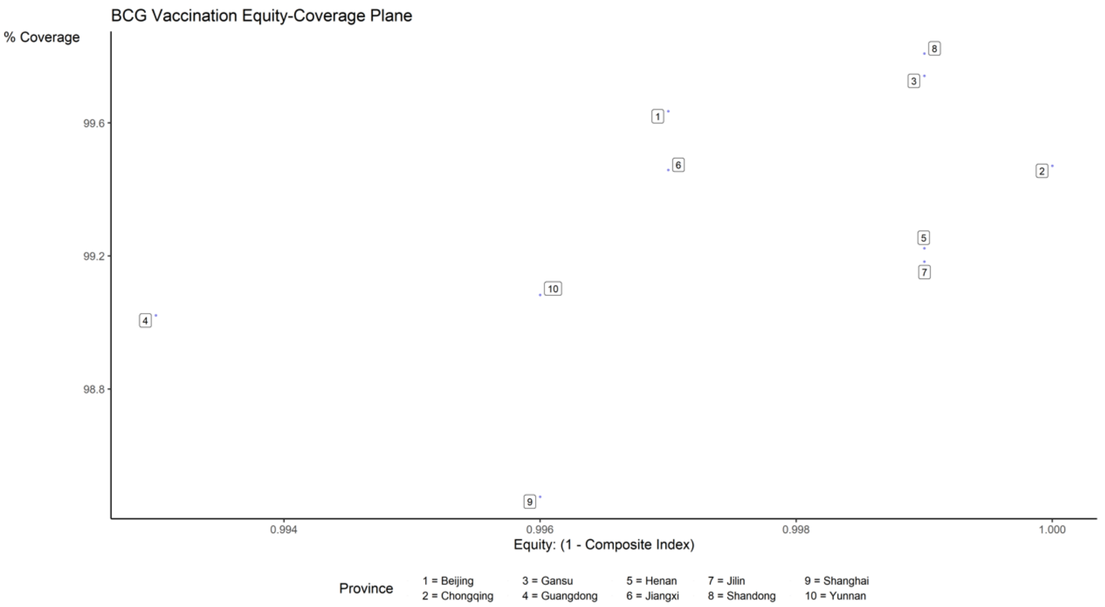

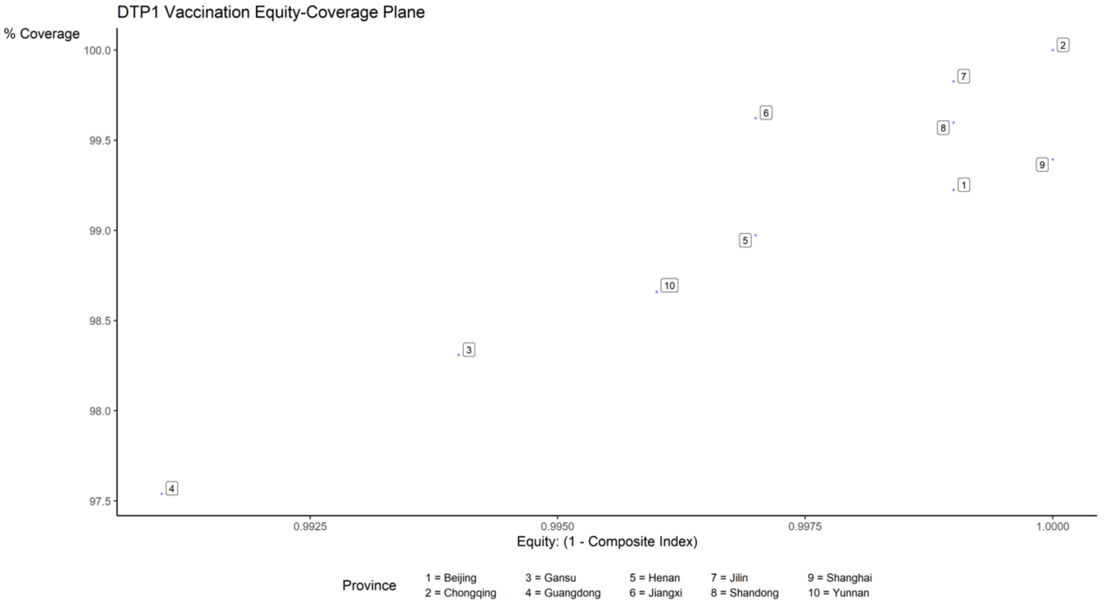

© 2022 Zhang H et al. *JAMA Network Open.*

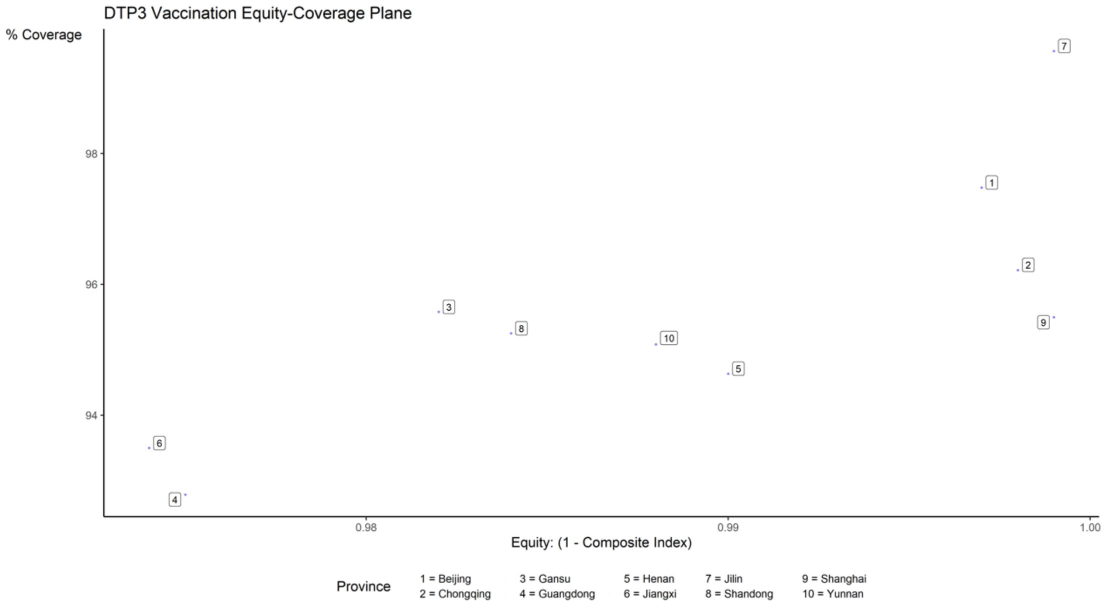

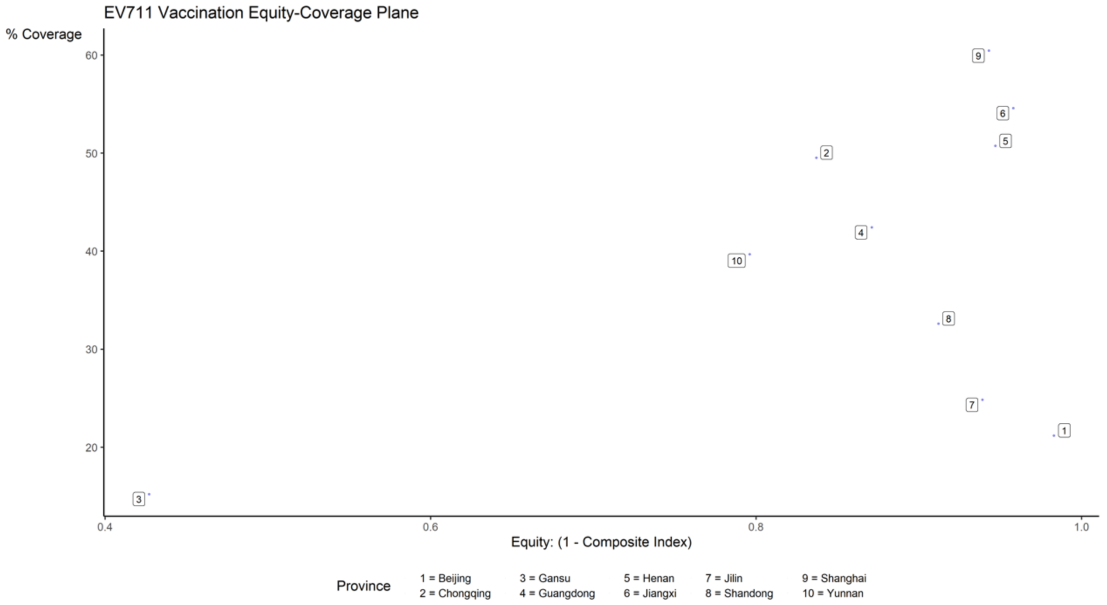

© 2022 Zhang H et al. *JAMA Network Open.*

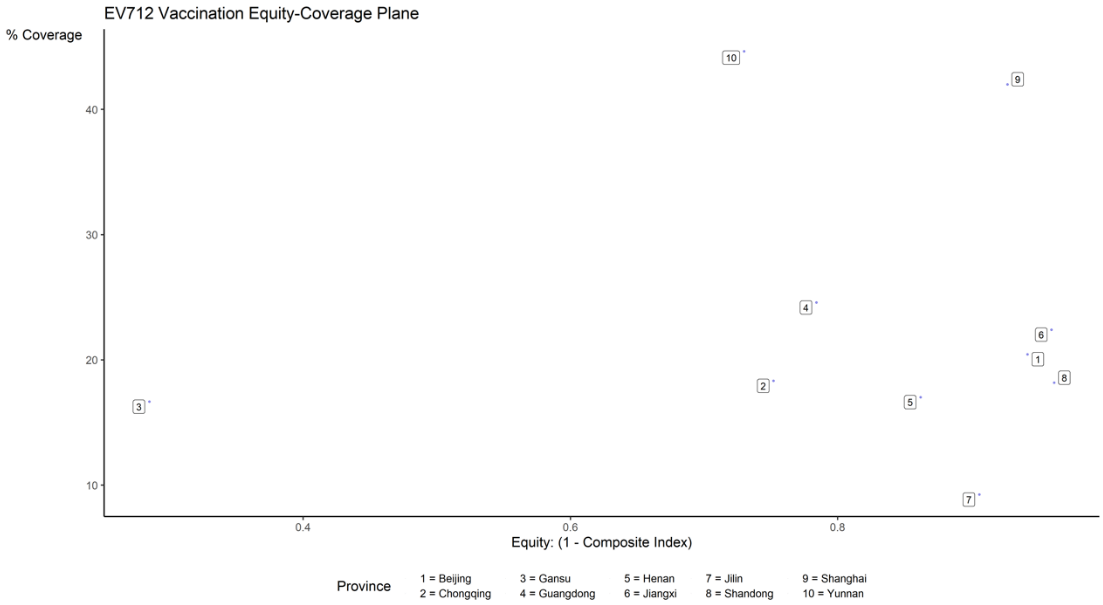

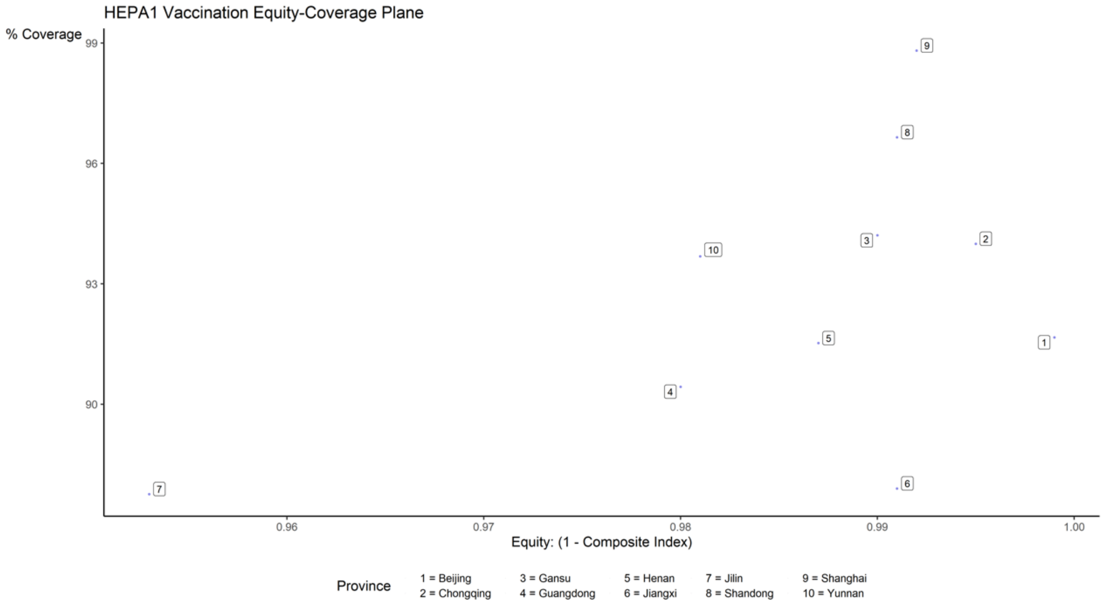

© 2022 Zhang H et al. *JAMA Network Open.*

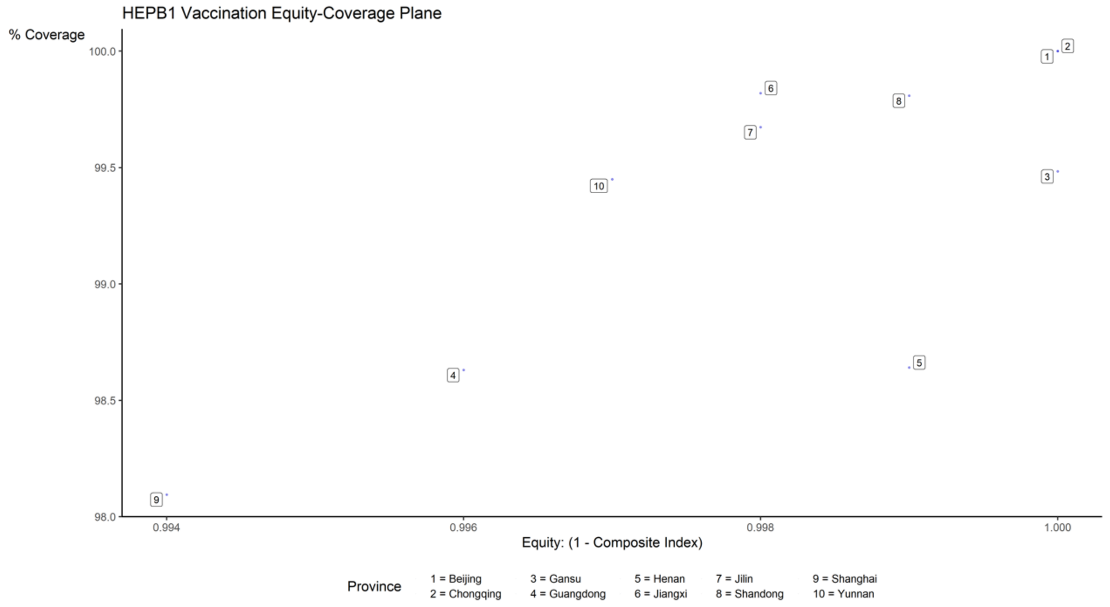

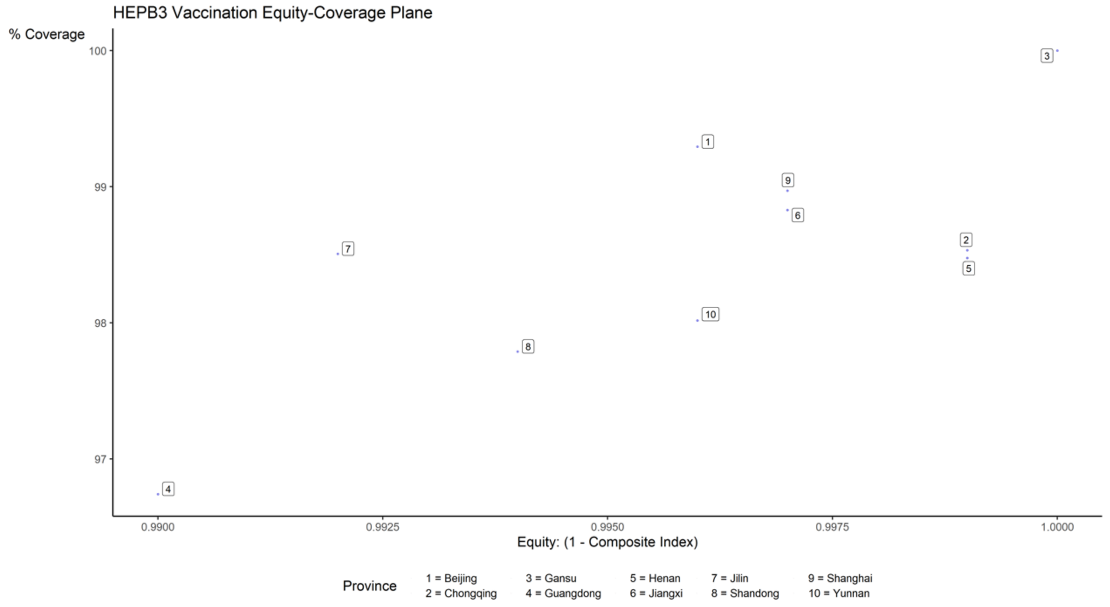

© 2022 Zhang H et al. *JAMA Network Open.*

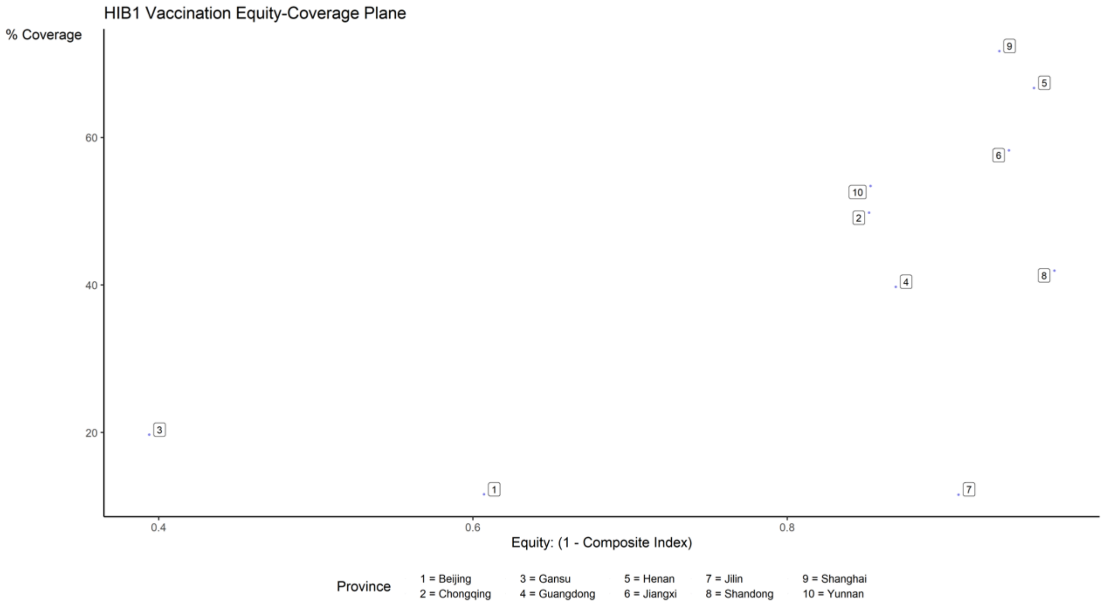

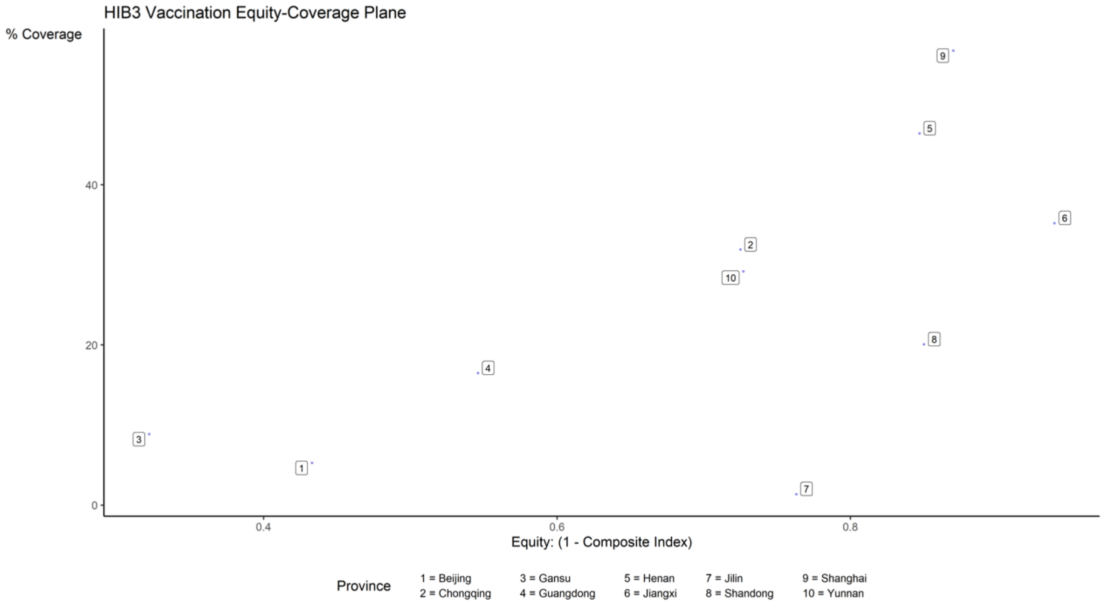

© 2022 Zhang H et al. *JAMA Network Open.*

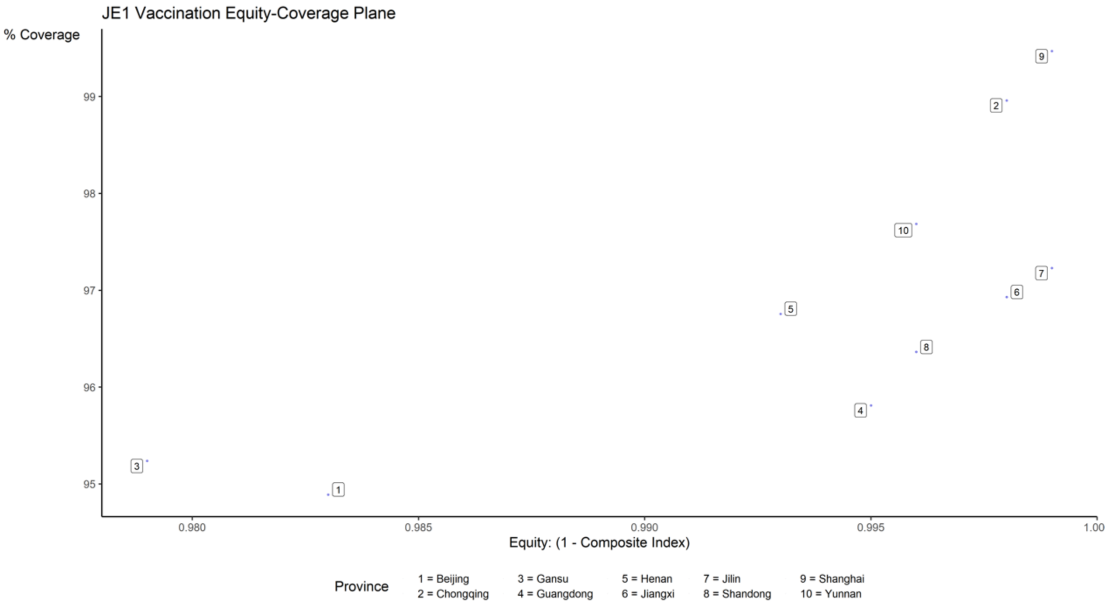

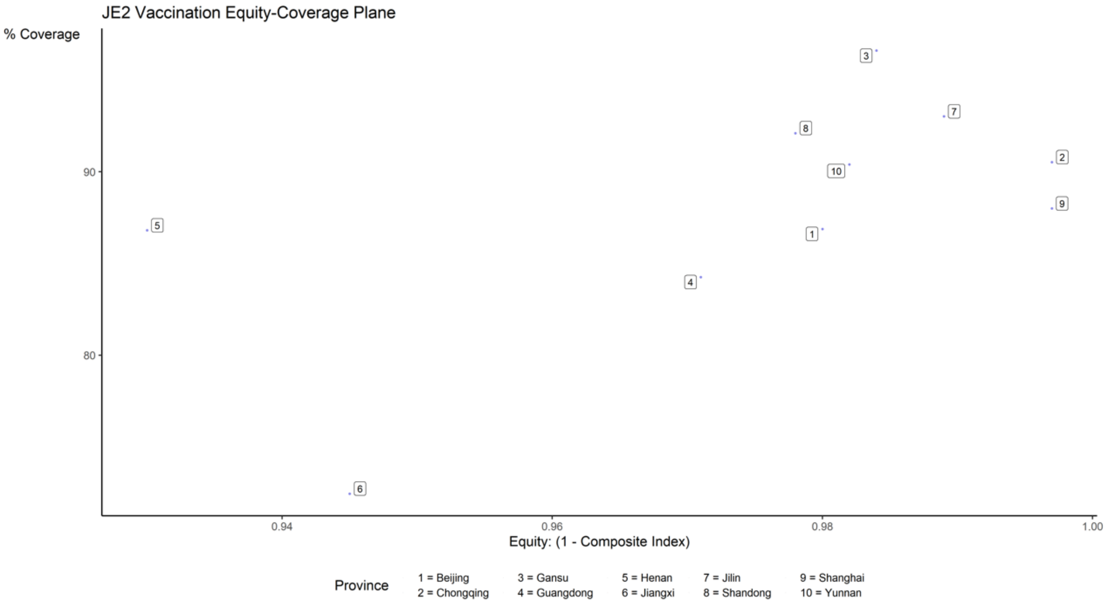

© 2022 Zhang H et al. *JAMA Network Open.*

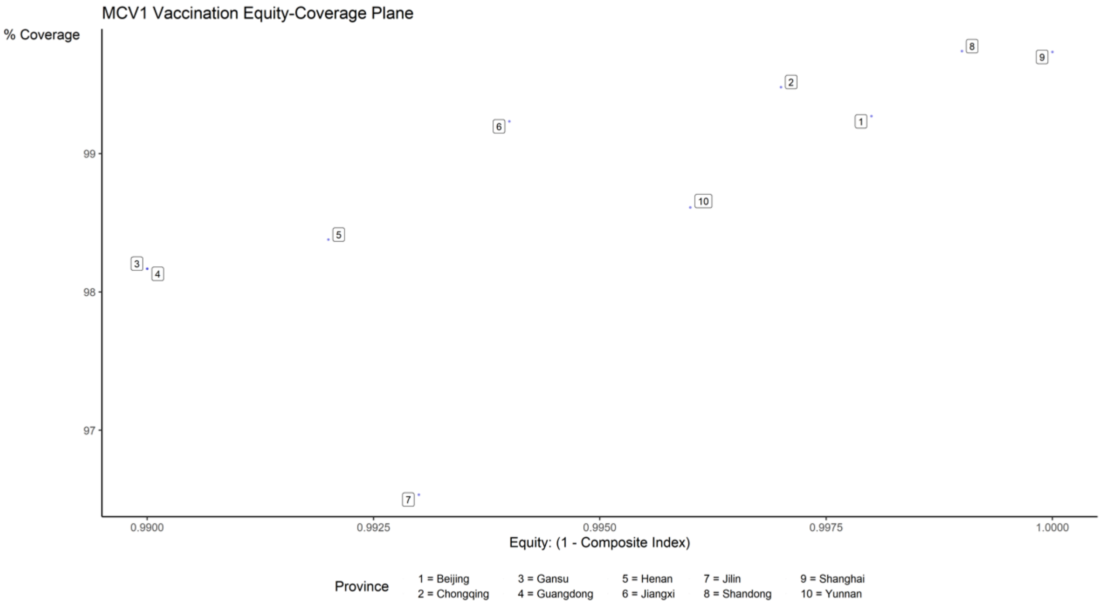

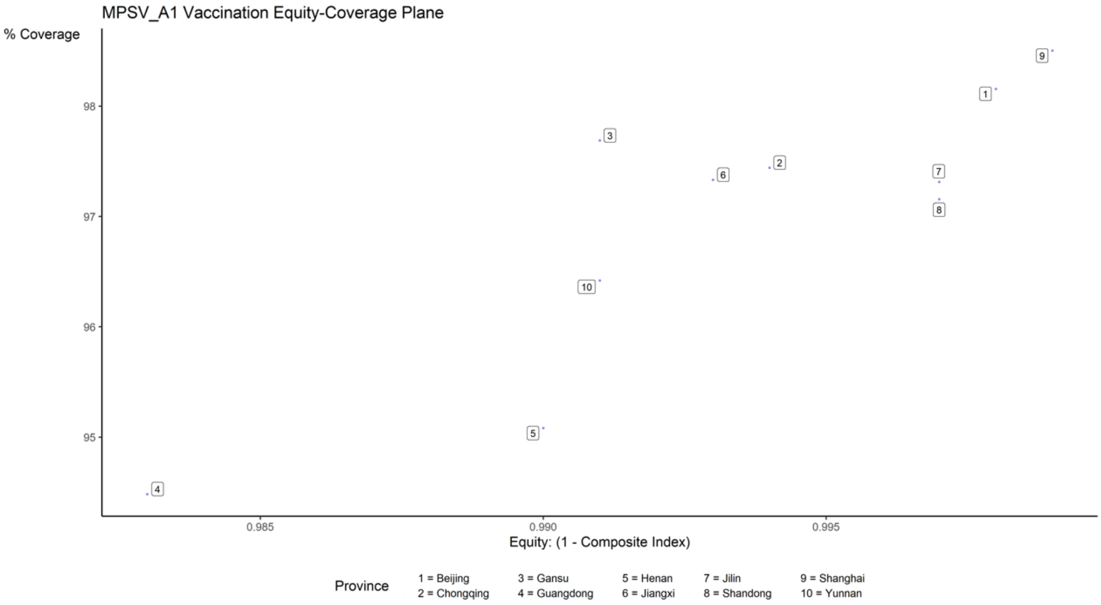

© 2022 Zhang H et al. *JAMA Network Open.*

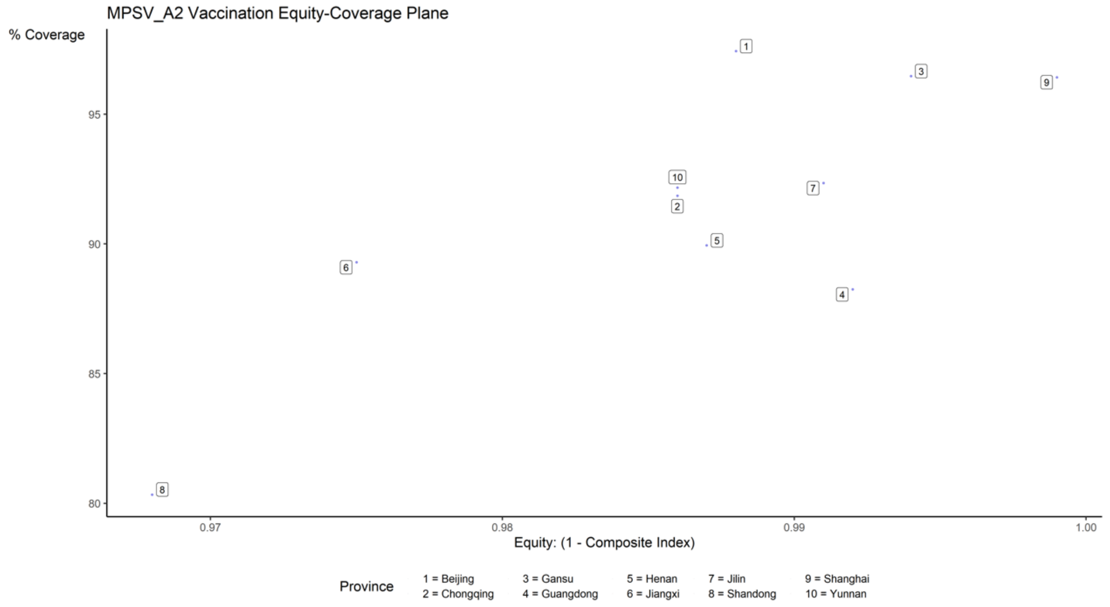

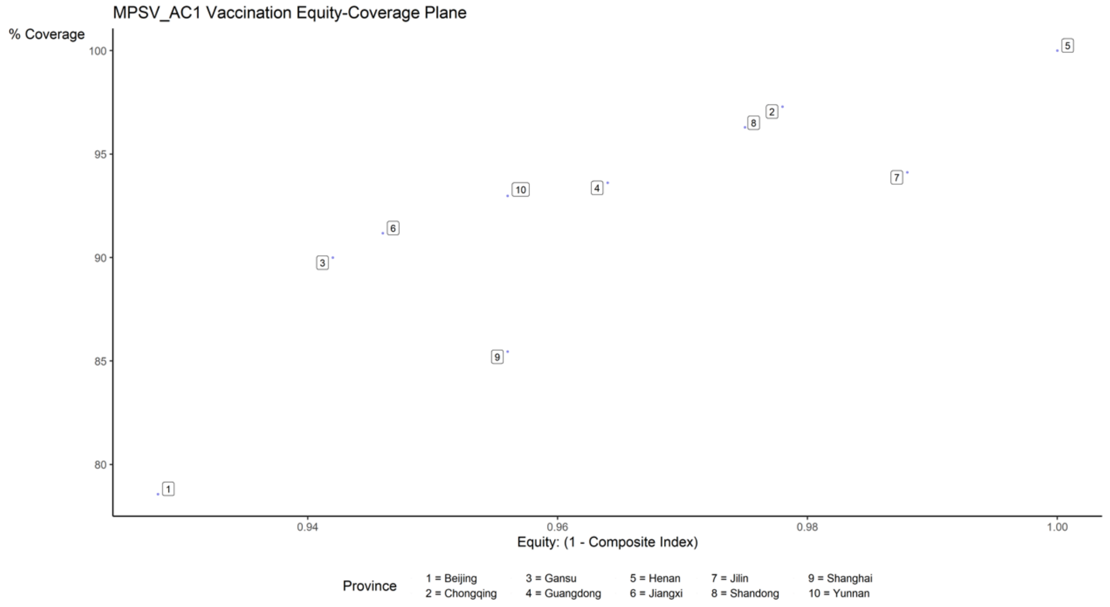

© 2022 Zhang H et al. *JAMA Network Open.*

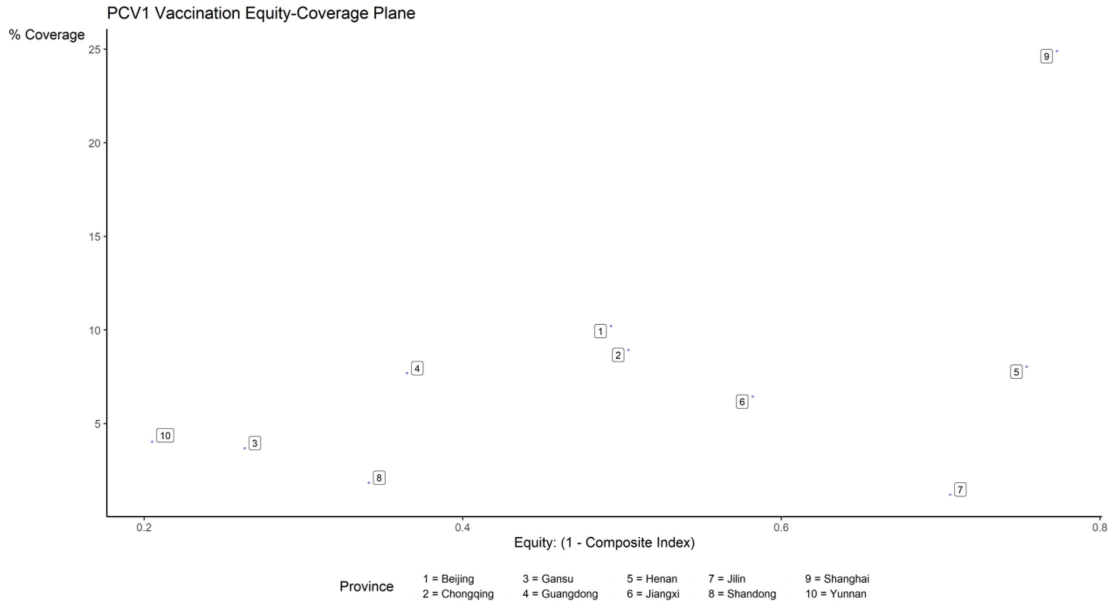

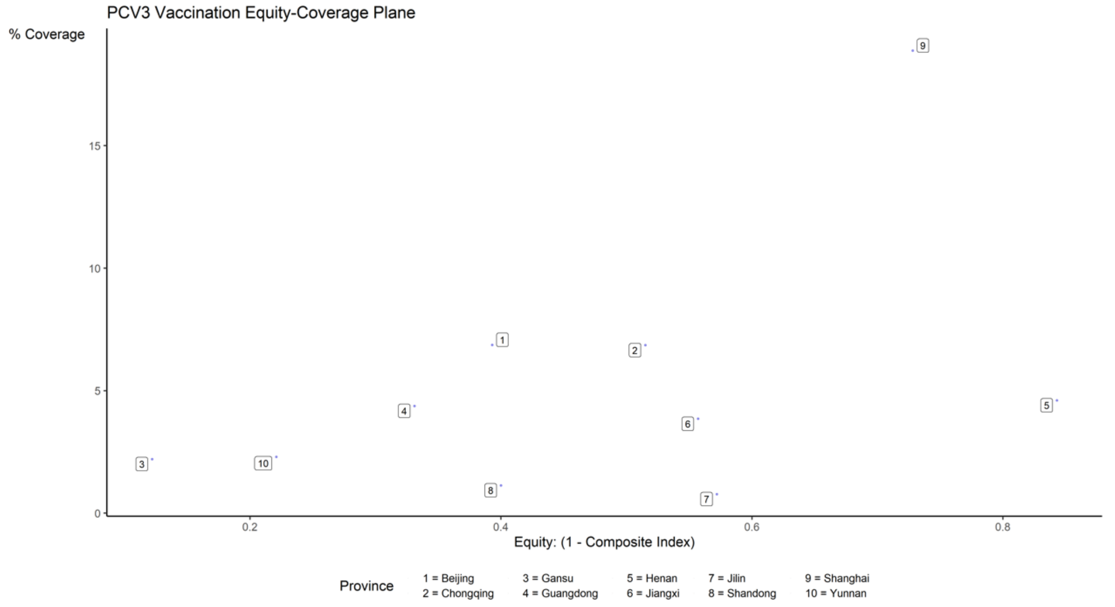

© 2022 Zhang H et al. *JAMA Network Open.*

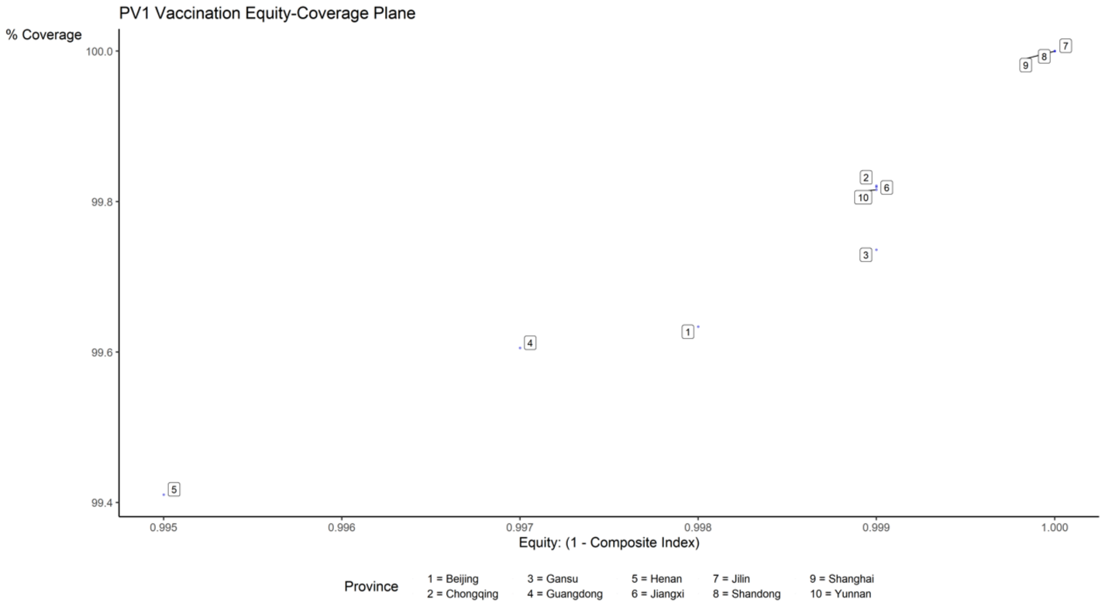

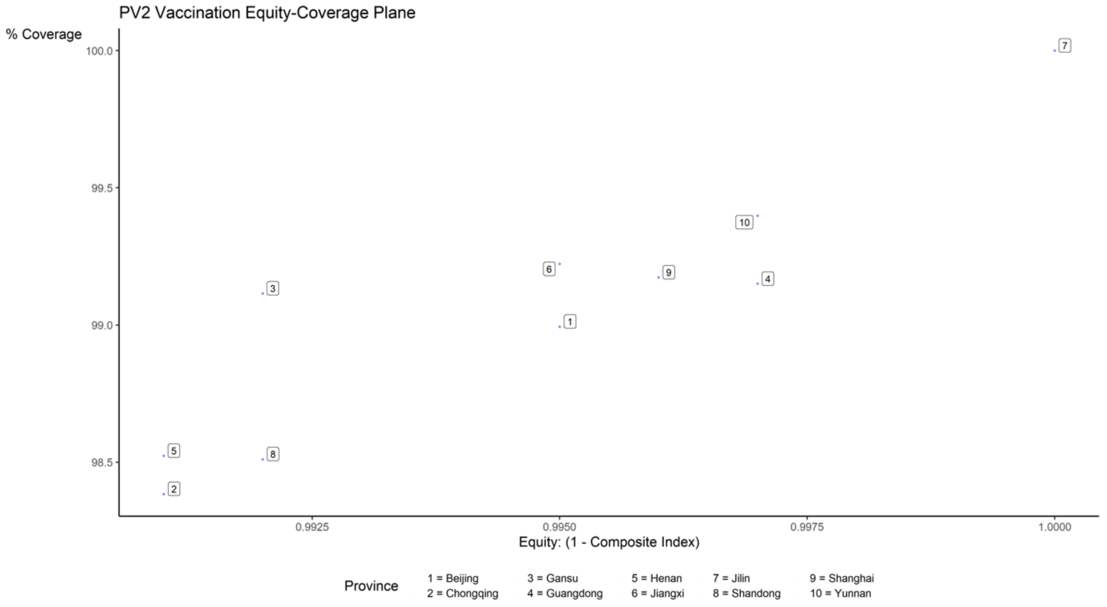

© 2022 Zhang H et al. *JAMA Network Open.*

PV3 Vaccination Equity-Coverage Plane

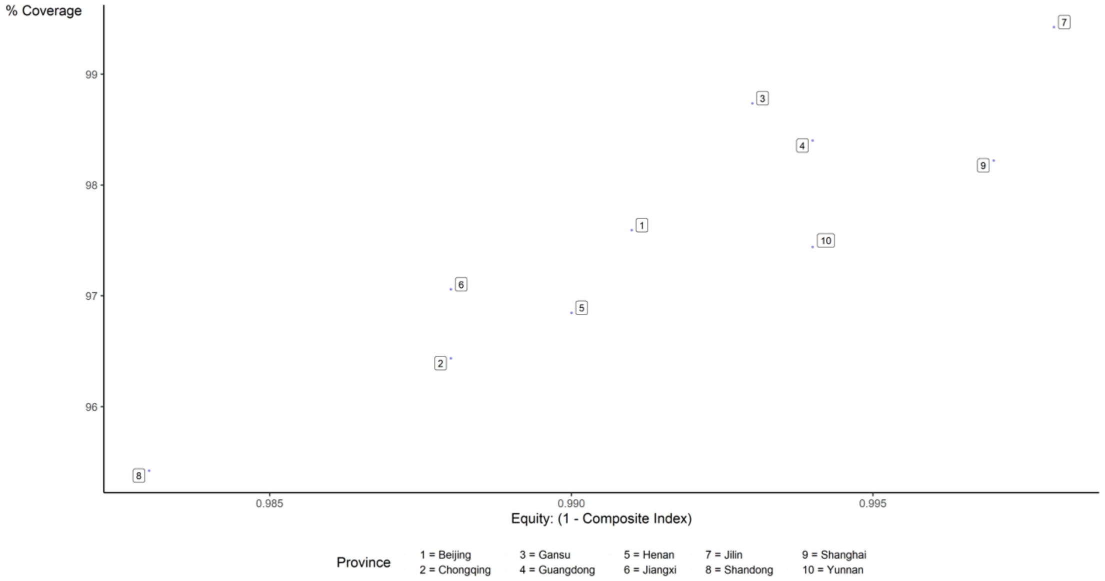

ROTA1 Vaccination Equity-Coverage Plane

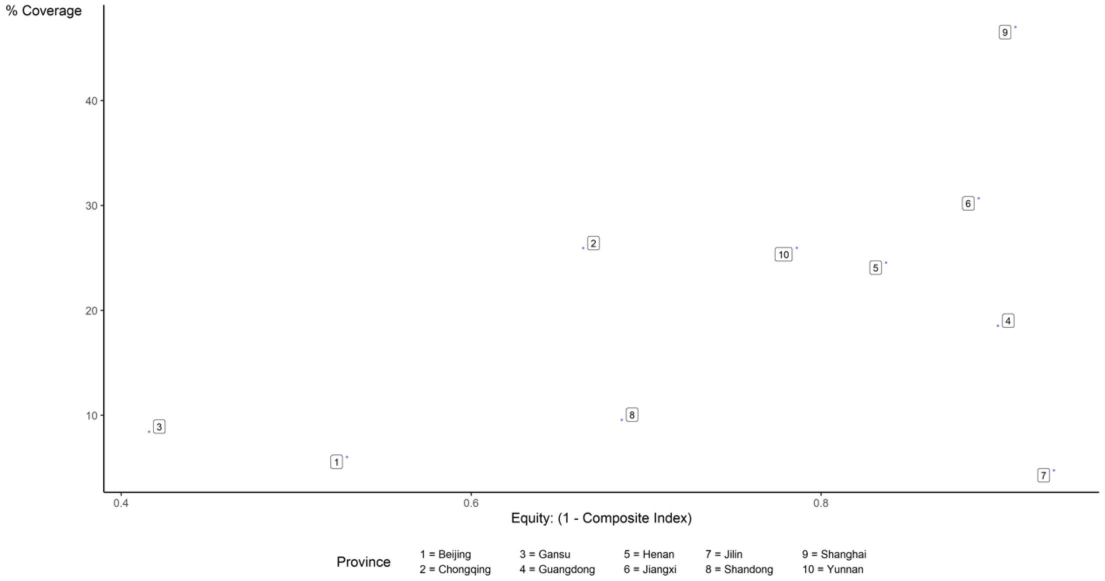

© 2022 Zhang H et al. *JAMA Network Open.*

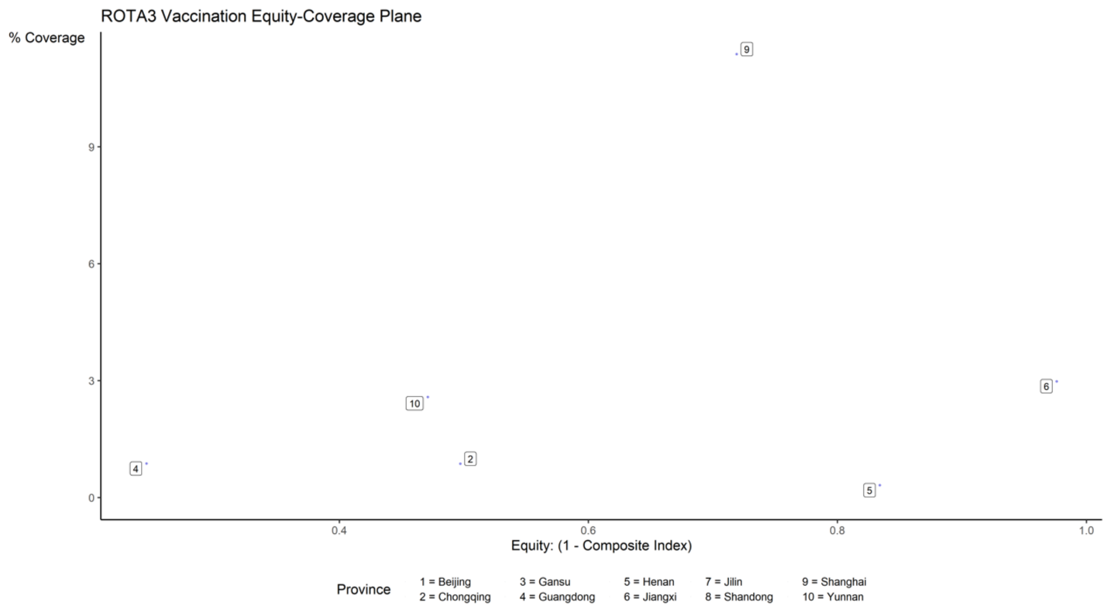

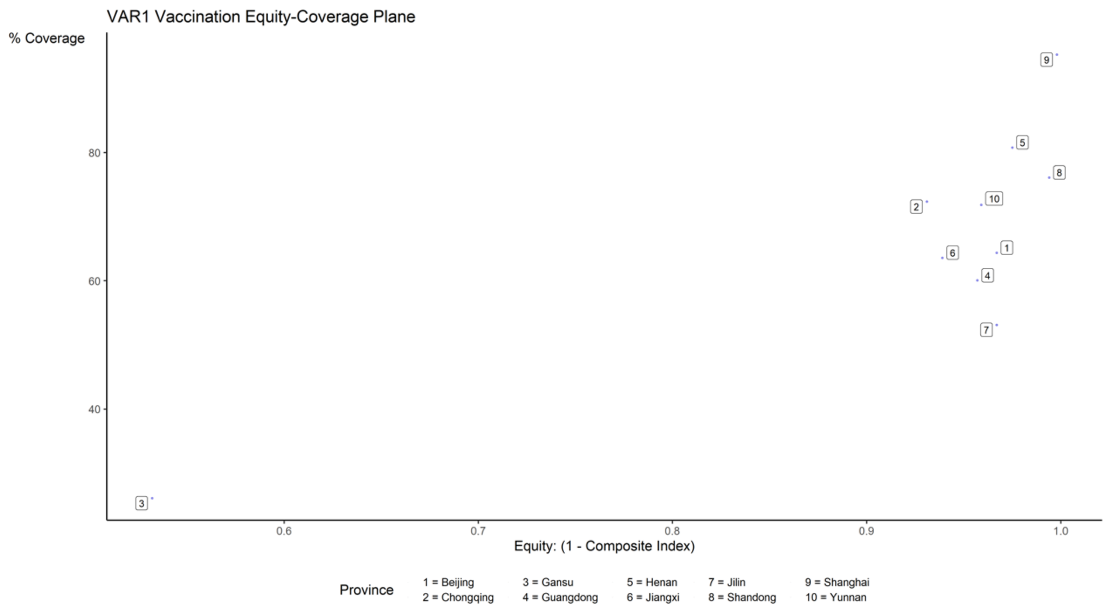

© 2022 Zhang H et al. *JAMA Network Open.*

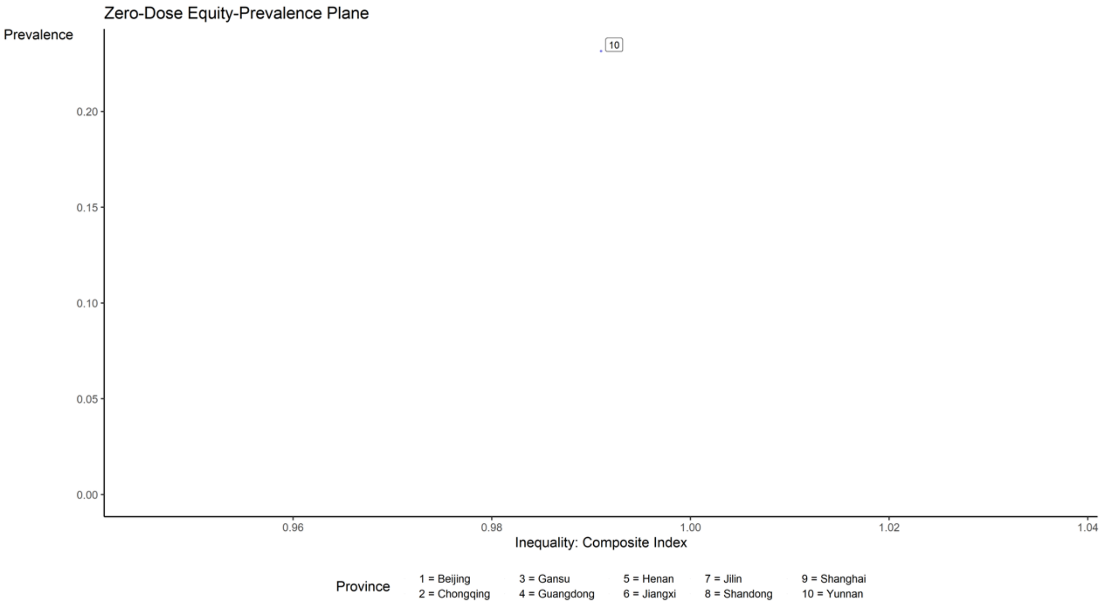

© 2022 Zhang H et al. *JAMA Network Open.*

eFigure 4. Decomposition of Vaccine Inequity

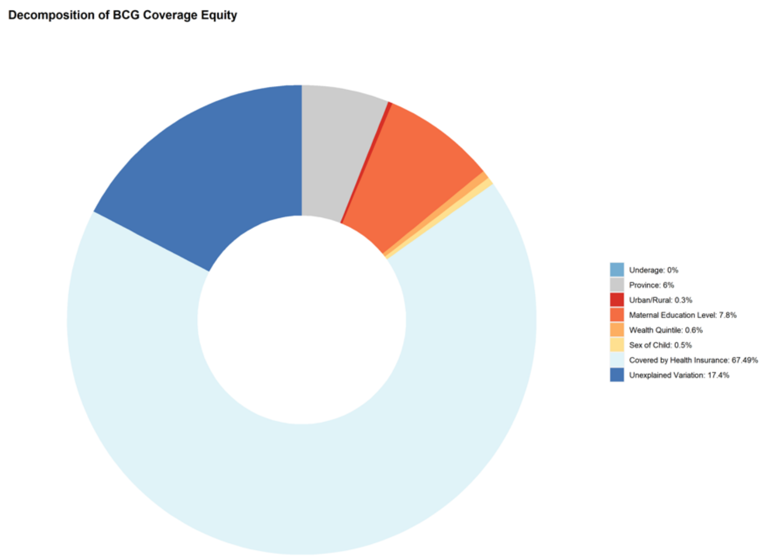

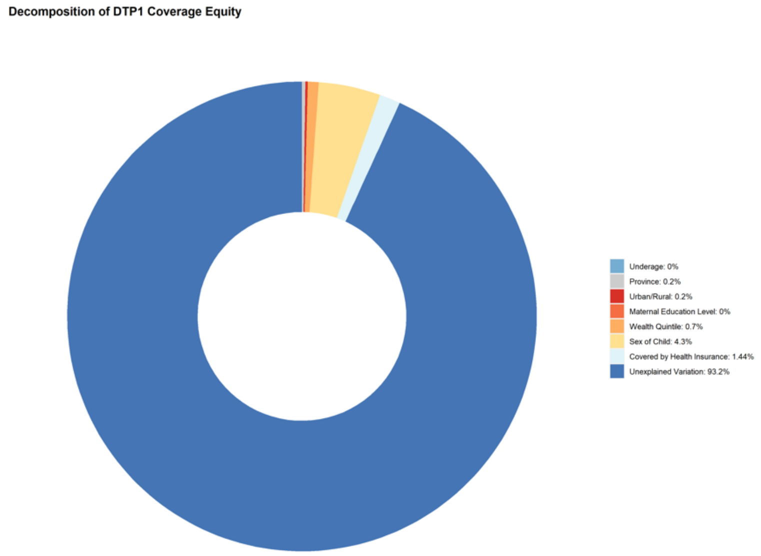

© 2022 Zhang H et al. *JAMA Network Open.*

Decomposition of DTP3 Coverage Equity

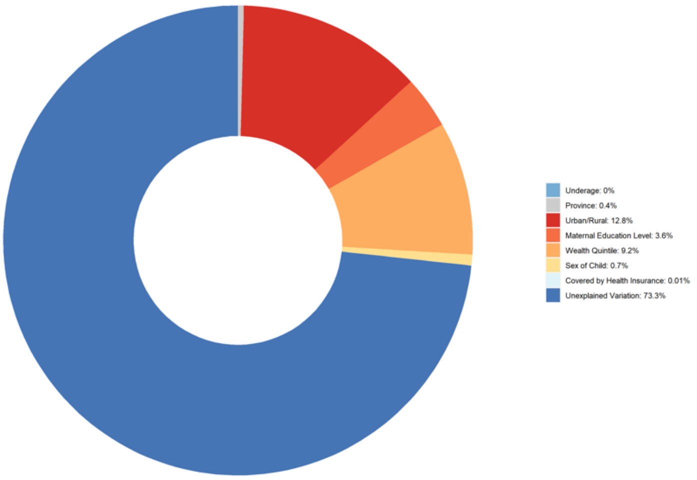

Decomposition of EV711 Coverage Equity

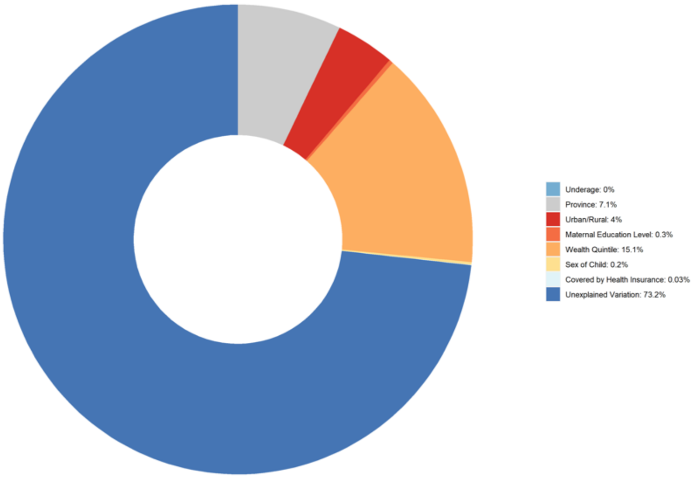

© 2022 Zhang H et al. *JAMA Network Open.*

Decomposition of EV712 Coverage Equity

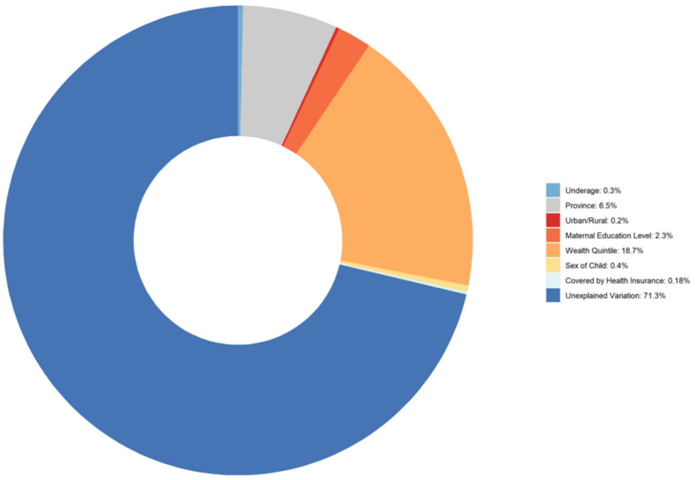

Decomposition of Equity in Fully NIP Immunized for Age Status

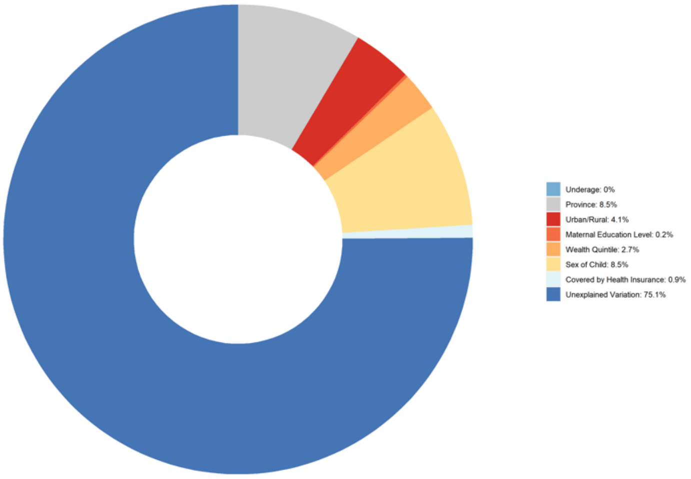

© 2022 Zhang H et al. *JAMA Network Open.*

Decomposition of HEPA1 Coverage Equity

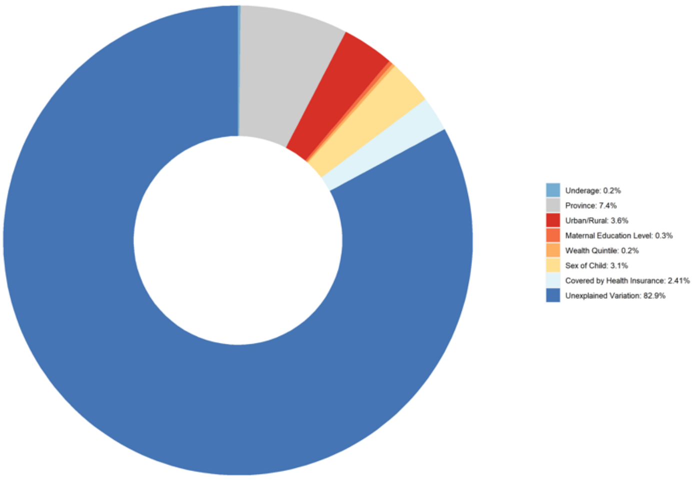

Decomposition of HEPB1 Coverage Equity

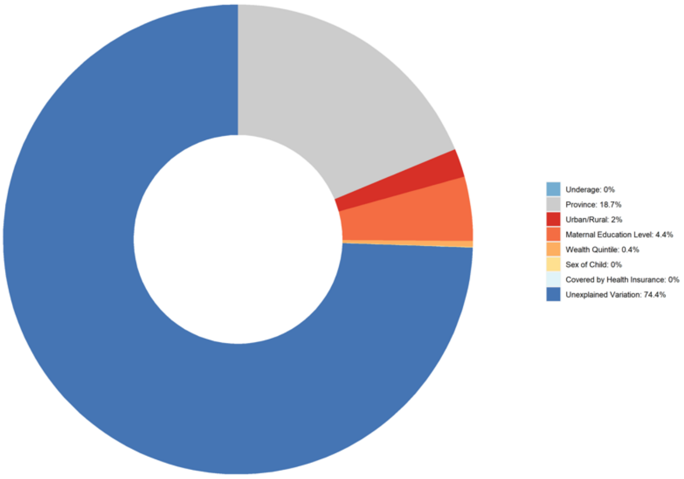

© 2022 Zhang H et al. *JAMA Network Open.*

Decomposition of HEPB3 Coverage Equity

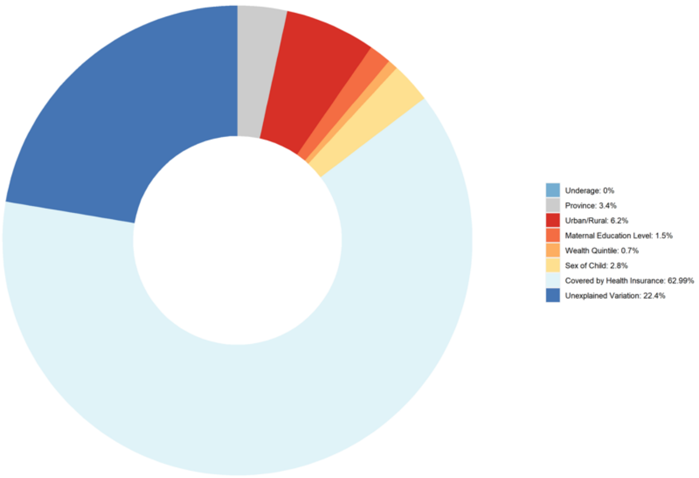

Decomposition of HIB1 Coverage Equity

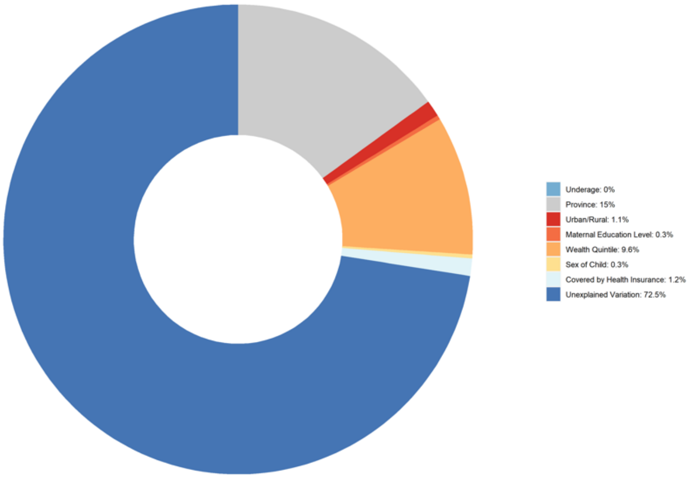

© 2022 Zhang H et al. *JAMA Network Open.*

Decomposition of HIB3 Coverage Equity

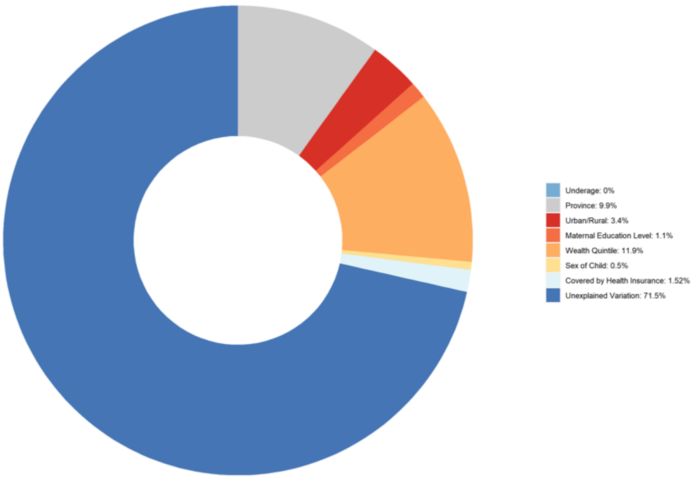

Decomposition of JE1 Coverage Equity

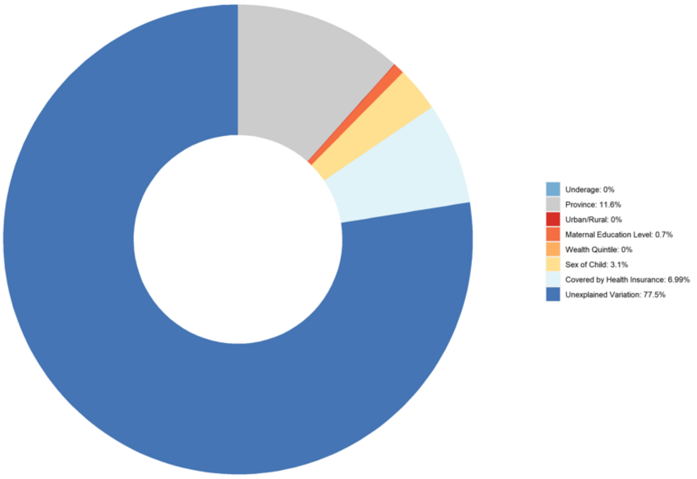

© 2022 Zhang H et al. *JAMA Network Open.*

Decomposition of JE2 Coverage Equity

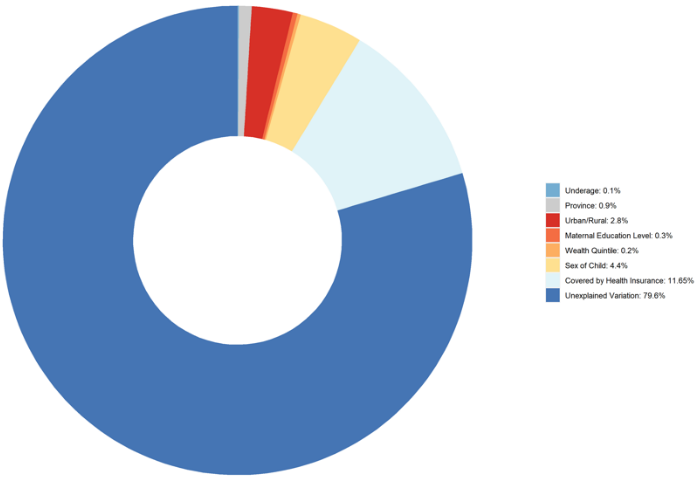

Decomposition of MCV1 Coverage Equity

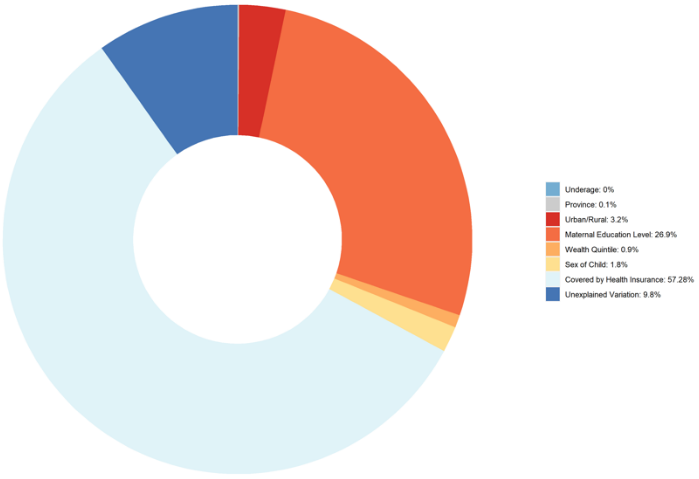

© 2022 Zhang H et al. *JAMA Network Open.*

Decomposition of MPSV_A1 Coverage Equity

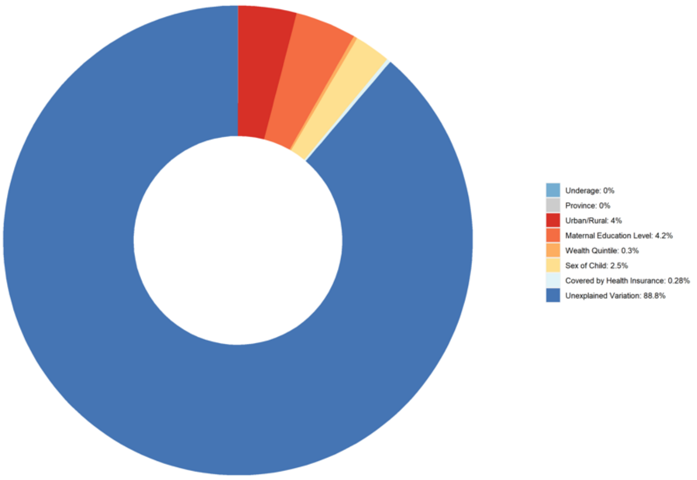

Decomposition of MPSV_A2 Coverage Equity

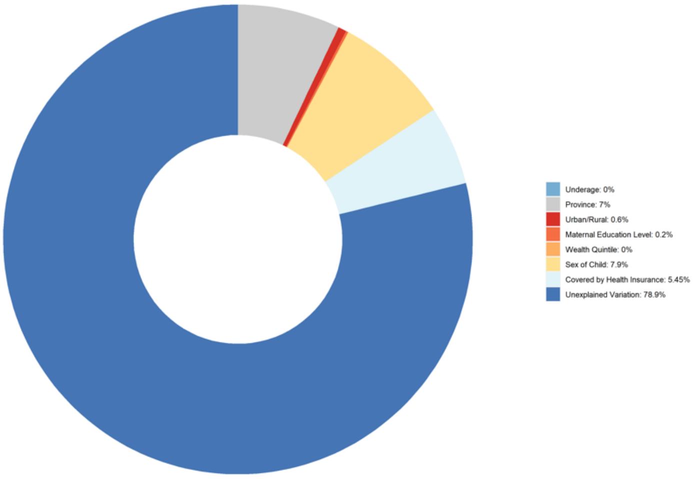

© 2022 Zhang H et al. *JAMA Network Open.*

Decomposition of MPSV_AC1 Coverage Equity

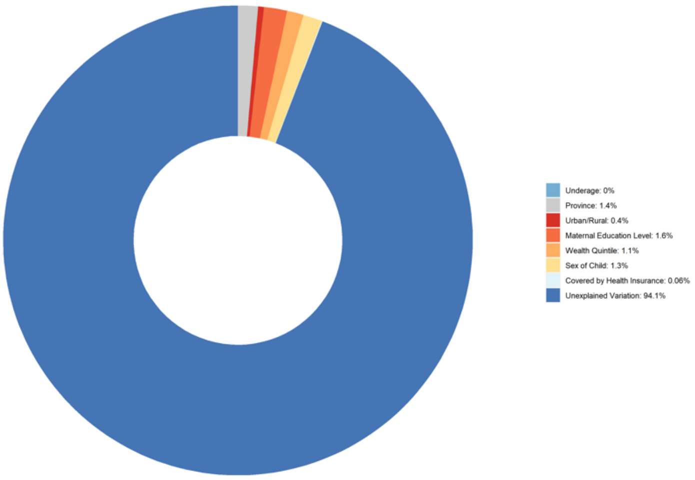

Decomposition of PCV1 Coverage Equity

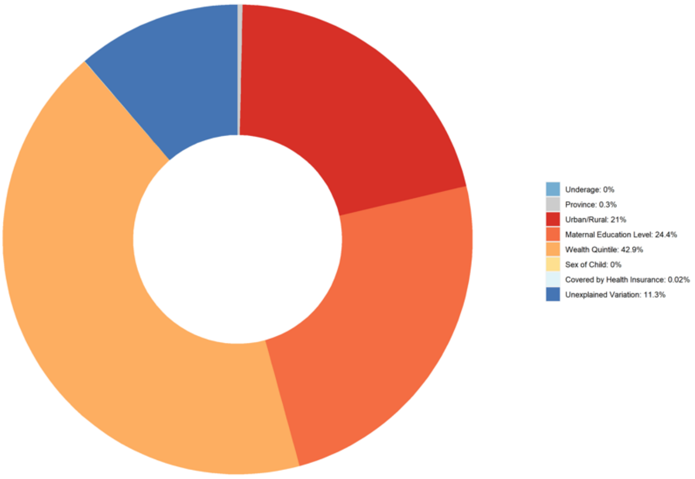

© 2022 Zhang H et al. *JAMA Network Open.*

Decomposition of PCV3 Coverage Equity

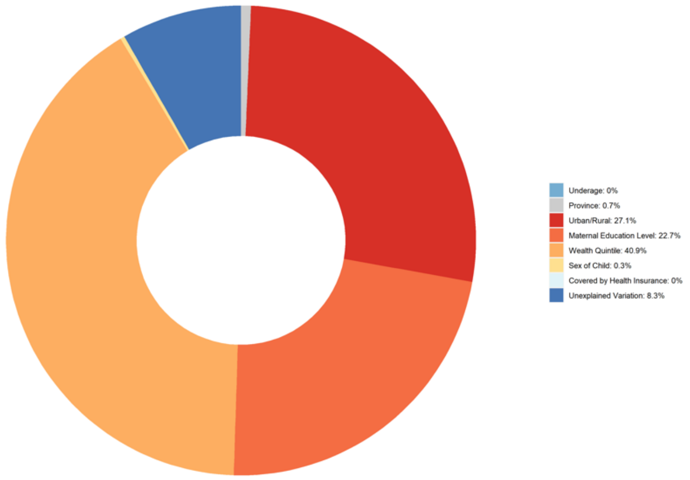

Decomposition of PV1 Coverage Equity

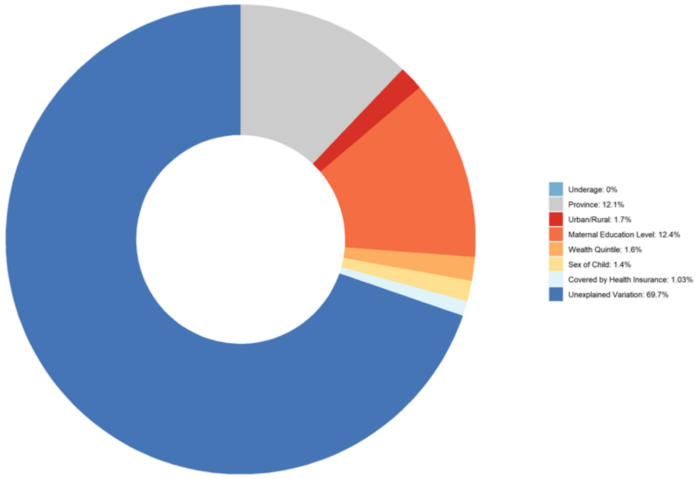

© 2022 Zhang H et al. *JAMA Network Open.*

Decomposition of PV2 Coverage Equity

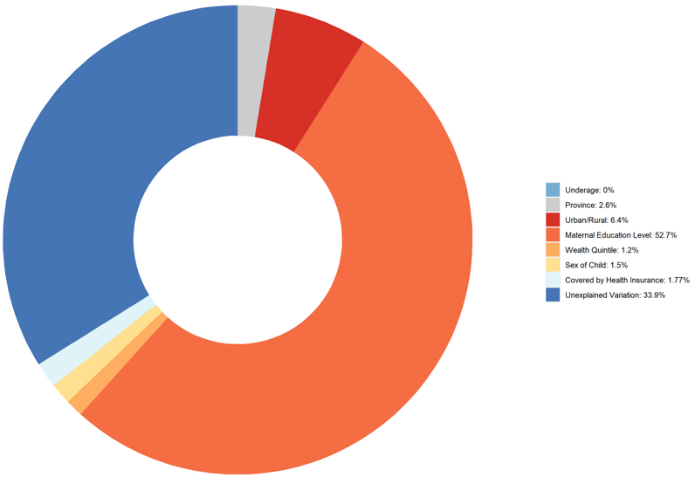

Decomposition of PV3 Coverage Equity

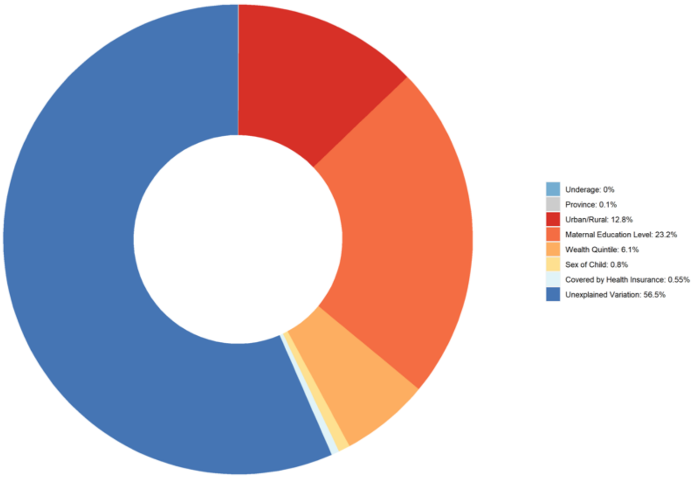

© 2022 Zhang H et al. *JAMA Network Open.*

Decomposition of ROTA1 Coverage Equity

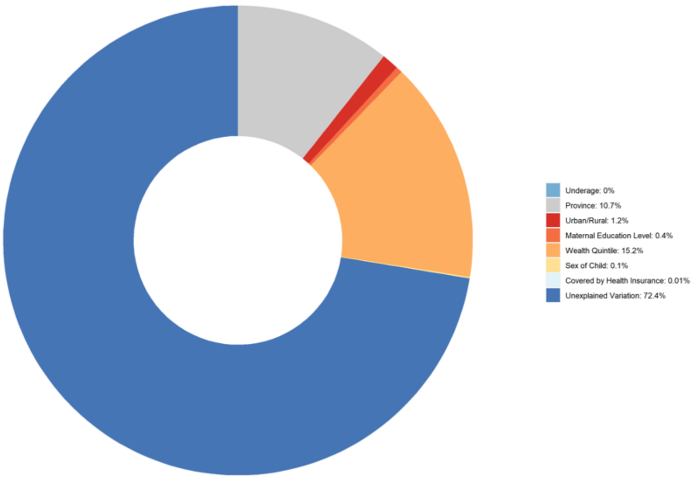

Decomposition of ROTA3 Coverage Equity

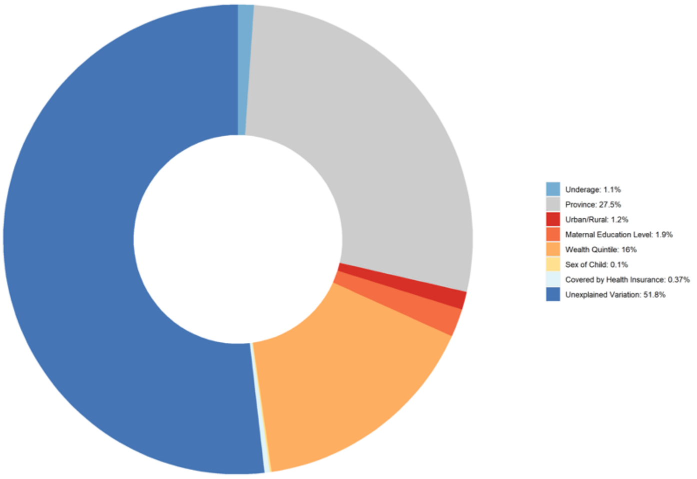

© 2022 Zhang H et al. *JAMA Network Open.*

Decomposition of VAR1 Coverage Equity

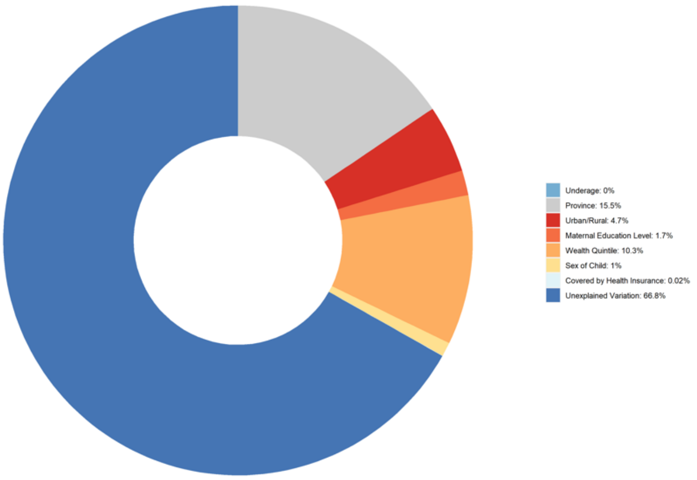

Decomposition of Zero-Dose Inequity

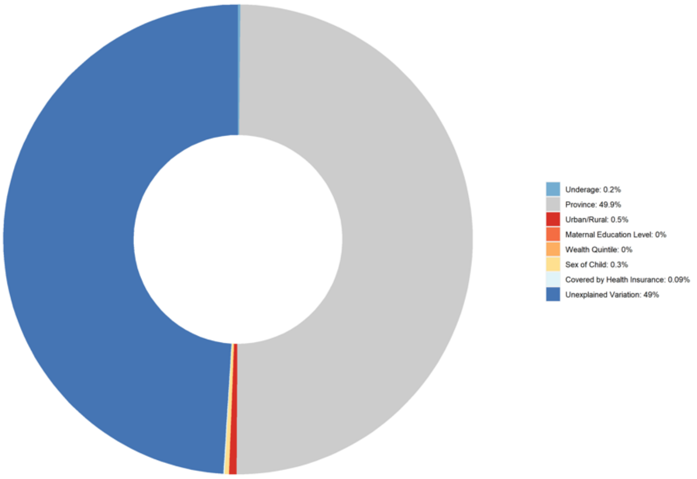

© 2022 Zhang H et al. *JAMA Network Open.*
