# Supplementary material for: Coverage and Equity of Childhood Vaccines in China
Source: JAMA Netw Open. 2022 Dec 9;5(12):e2246005. doi: 10.1001/jamanetworkopen.2022.46005 (PMC9856225; doi:10.1001/jamanetworkopen.2022.46005)
Supplement: Supplement 2. — Data Sharing Statement [file jamanetwopen-e2246005-s002.pdf]

Data Sharing Statement

Zhang H, Lai X, Mak J, et al. Coverage and equity of childhood vaccines in China. *JAMA Netw Open*. 2022;5(12):e2246005. doi:10.1001/jamanetworkopen.2022.46005

Data
Data available: No

Additional Information
Explanation for why data not available: Data for childhood immunization records and socioeconomic characteristics are not sharable according to the respondents' consent agreement.

© 2022 Zhang H et al. *JAMA Network Open*.
